# Bayesian model discovery for reverse-engineering biochemical networks from data

**DOI:** 10.1371/journal.pone.0355545

**Published:** 2026-08-20

**Authors:** Andreas Christ Sølvsten Jørgensen, Marc Sturrock, Atiyo Ghosh, Vahid Shahrezaei

**Affiliations:** 1 Department of Mathematics, Imperial College London, London, United Kingdom; 2 I-X Centre for AI In Science, Imperial College London, London, United Kingdom; 3 Laboratoire des Sciences du Climat et de l’Environnement, CEA CNRS UVSQ, Orme des Merisiers, Gif-sur-Yvette, France; 4 Department of Physiology and Medical Physics, Royal College of Surgeons in Ireland, Dublin, Ireland; Instituto Nacional de Medicina Genomica, MEXICO

## Abstract

Reverse engineering gene regulatory networks from gene expression data is a challenging inference task. A related problem in computational systems biology is identification of signalling networks that perform particular functions, such as adaptation. Indeed, for many research questions, there is an ongoing need for efficient inference algorithms that can identify the simplest model, from among a larger set of inter-related models, that best explains empirical observations. To this end, we introduce Sparse Likelihood-free Inference using Gibbs sampling (SLInG), a Bayesian sparse likelihood-free inference method. SLInG provides an efficient sampling method for Approximate Bayesian Computation with sparsity-inducing hierarchical priors that is widely applicable for any simulation-based model discovery task. We first apply SLInG to linear sparse regression problem using a classic dataset, before focusing on applications to biochemical network model discovery. We demonstrate that SLInG can reverse engineer stochastic gene regulatory networks from single-cell data with high accuracy, outperforming state-of-the-art correlation-based methods. Furthermore, we show that SLInG can successfully identify signalling networks that execute adaptation. Sparse hierarchical Bayesian inference thus provides a versatile and powerful tool for model discovery in systems biology and beyond.

## Introduction

In systems biology and indeed many areas of science and engineering, one is interested in using quantitative data to infer an underlying mechanistic model governing a particular phenomenon. This statistical inference task is commonly known in the literature as equation discovery or *model discovery*, e.g., [1–5]. Another challenge is that many of the underlying mathematical models are too complex to have a tractable likelihood but can be simulated for specific parameter values. In recent years, a broad class of inference methods known as likelihood-free or simulation-based inference methods have been developed to tackle this task [6]. A well-known class of Bayesian simulation-based inference methods is Approximate Bayesian Computation (ABC) [7–10] that has been applied to different types of real-world data from cancer to stellar physics, e.g., [11–13]. In essence, by comparing experimental data to a large set of simulations conducted over various parameters sampled from prior distributions, researchers can determine the ranges of parameter values that can approximately recover observations. While a wide range of ABC methods are developed for parameter inference and model selection (among a small number of alternative models), flexible and efficient ABC approaches for model discovery are still lacking.

To clarify the distinctions between parameter inference, model selection, and model discovery, let us look at an example. Consider the case of fitting time series (or snapshot) data to ordinary or partial differential equations (ODEs/PDEs). The simplest situation exemplifying parameter inference is when functional form of the equations is known, and we are only trying to infer parameters of these models cf. [14–17]. However, in addition to having to constrain model parameter values, one might not *a priori* know what mechanisms to include in models or how to capture system dynamics correctly. If we are only choosing among a small number of specific candidate ODE/PDE models, the inference task is a classic model selection, and one can use, for example, ABC model selection [7,10]. Finally, equation discovery or model discovery deals with a scenario in which we are not limited to a small number of alternative models and do not know what terms to include in the ODEs/PDEs. Hence, model discovery can be seen as a large-scale model selection task between closely related models. One approach is to use a predefined library of possible mathematical terms (with their own associated unknown parameters) and to define the most general and complex model, while simpler models (so-called submodels) are constructed by removing a subset of these terms (by setting their coefficients to zero). Therefore, the task of equation discovery is closely related to sparse regression and variable selection [18,19]. Here, the term sparsity refers to reducing the number of non-zero coefficients or parameters. Some pioneering equation discovery approaches have indeed been developed in this way in the context of ODE/PDE discovery from time-series data such as SINDy [1].

In this paper, we present an efficient and versatile Bayesian model discovery approach based on ABC that is applicable to any simulation-based model discovery task, including stochastic models. Our algorithm, which we name Sparse Likelihood-free Inference using Gibbs sampling (SLInG), uses hierarchical sparsity-inducing priors and Gibbs sampling similar to the Bayesian LASSO (least absolute shrinkage and selection operator) by Park & Casella that uses a Gibbs sampling algorithm [18,20,21] and is specifically based on an ABC Gibbs algorithm by Turner & Van Zandt [22]. Moreover, SLInG is placed among adaptive LASSO approaches, such as Sorted L-One Penalised Estimation (SLOPE) cf. [23,24] and references therein where each parameter has its own sparsity-inducing hyper-parameter. This property allows SLInG to autonomously adjust the level of sparsity invoked for each parameter in a data-dependent fashion. Unlike LASSO and SLOPE, our approach is not limited to linear models as demonstrated in our systems biology applications.

We present the application of SLInG to three case studies. In the Linear models section, we address a linear model of diabetes progression [18,25]. The model provides a simple and well-studied example, allowing us to highlight and test the basic assets of our sparse sampling algorithm.

In the Adaptive protein signalling networks section, we use our algorithm to identify protein signalling networks that achieve biochemical adaptation, i.e., we look for network topologies with certain response properties [26–32]. Many studies in systems biology focus on understanding and mapping topologies that execute particular biological functions, such as adaptation. The search for efficient algorithms to perform this inference task is ongoing, e.g., [28,33–37].

Finally, in the Stochastic regulatory genetic networks section, we employ our algorithm to identify links in stochastic gene regulatory networks from (synthetic) data, i.e., we seek to infer the specific network topology that underlies the data. This reverse engineering task poses a challenging problem in systems biology. Moreover, many of the existing methods for performing this reverse engineering are not based on mechanistic models and rely only on the construction of a correlation network [38]. We show that our algorithm can compete with other algorithms specifically tailored with this inference task in mind [39,40].

## Results

We introduce SLInG for inferring simple (sparse) models describing a system using a Bayesian likelihood-free approach. In short, SLInG is a Gibbs sampling algorithm for ABC with sparsity-inducing priors that aims to minimise the distance (*d*) between the data and model predictions while discarding superfluous parameters. This concept is relevant to the general problem of simulation-based model discovery. Before demonstrating the applicability and performance of SLInG in three case studies in the Linear models, Adaptive protein signalling networks and Stochastic regulatory genetic networks sections, we present the SLInG algorithm in detail in the Algorithm section.

### Algorithm

**Fig 1:**
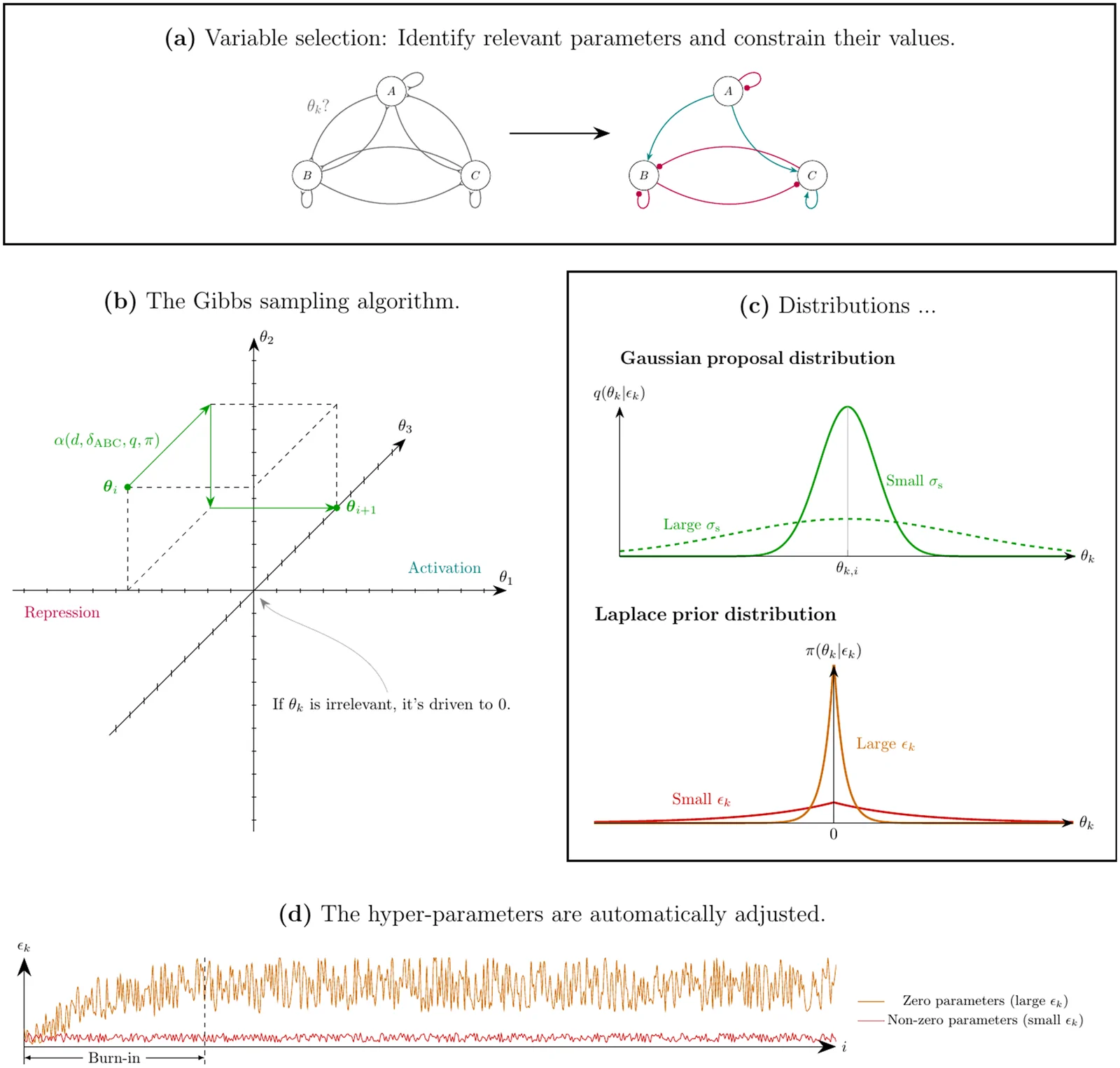
Schematic overview of the different components of SLInG. (a) Summary of sparse inference tasks exemplified through gene regulatory networks: We discard irrelevant links and constrain the properties of the remaining links. (b) To perform one step in the Markov chain starting from the parameter values at the *i*th iteration ($\theta_{i}$), the Gibbs sampler takes *K* perpendicular steps through the *K*-dimensional parameter space updating one parameter at a time. For each perpendicular step, one simulation is computed. The acceptance probability (α) at each perpendicular step depends on the parameter $\delta_{ABC}$ (that sets the overall level of sparsity), the proposal distribution *q*, the prior π (and hence the hyper-parameters, ϵ), and the distance (*d*) between the predictions and the data. (c) The perpendicular steps are sampled from proposal distributions with adaptive width ($\sigma_{s}$). Sparsity is imposed through parameter-specific priors. (d) The parameter-specific hyper-parameters (ϵ) of the priors are constantly updated. Each line in panel (d) corresponds to a single hyper-parameter $\epsilon_{k}$ as it evolves across iterations of the Gibbs sampler: lines associated with parameters that the chain is driving towards zero rise towards large values of $\epsilon_{k}$ (strong shrinkage), while lines associated with parameters supported by the data settle at small values of $\epsilon_{k}$ (relaxed shrinkage).

Consider a general model that we can simulate with *K* parameters $\theta =(\theta_{1},\theta_{2},...,\theta_{k})$. Moreover, we have a set of observations or data that the model should be able to explain. The inference problem tackled by SLInG is to use sparsity-inducing priors to approximate the posterior distribution over the parameter space given the data. This will result in discarding superfluous parameters (parameters that can be set to zero) and obtaining confidence intervals for the remaining parameters. This approach identifies the simplest sub-model(s) that can explain the data (Fig 1a).

Within Bayesian statistics, our goal of sparse inference, i.e., variable selection, can be achieved by introducing a variety of sparsity-inducing priors [19]. In the context of SLInG, we impose Laplace priors on the model parameters, e.g., [18,41] as done in Bayesian LASSO, a regression analysis employing the least absolute shrinkage and selection operator. Laplace prior’s sharp peak at zero and heavy tails encourage parameter values to be either exactly zero or to take non-zero values, making it well-suited for variable selection. This is the Bayesian analogue of the L1 regularisation term in classical LASSO. In contrast, Gaussian priors shrink parameters towards zero but do not drive them to be exactly zero. The priors themselves involve free parameters (ϵ), which implies that the problem can be phrased as a hierarchical model, i.e., ϵ are hyper-parameters. While it is commonplace to use the same hyper-parameters for all parameters, e.g., in LASSO [42], we assign a new hyper-parameter $\epsilon_{k}$ to each parameter, $\theta_{k}$. A similar approach is taken in the adaptive LASSO and SLOPE as mentioned in the introduction [21,24,43]. The use of parameter-specific hyper-parameters allows us to handle parameters of different orders of magnitude and to sift out redundant parameters without skewing the posterior distributions of the non-zero parameters. Thus, for a single parameter $\theta_{k}$, the Laplace distribution centred around zero is given by the probability density function


$$\begin{array}{c} \pi (\theta_{k}|\epsilon_{k})=\frac{\epsilon_{k}}{2}\exp\left(-\epsilon_{k}|\theta_{k}|\right), \end{array}$$ (1)

where $\epsilon_{k}$ is the corresponding hyper-parameter. In addition, our implementation provides the option to define a region of the parameter space to explore. Our algorithm thus employs truncated sparsity priors by combining equation (1) with uniform priors. We use truncated Laplace priors for all case studies presented in the paper.

To circumvent the intractable likelihood faced in many real-world applications, we substitute the likelihood in the acceptance rate of our Markov-Chain Monte Carlo approach with a kernel function $\psi (d(s_{sim},s_{obs})/\delta_{ABC})$ based on a distance measure $d(s_{sim},s_{obs})$ between some summary statistics (*s*) of the simulation and the data. Note that it is the use of a distance measure between the summary statistics, *d*, that places the algorithm within the realm of ABC methods [10]. The distance measure and summary statistics used is problem specific. In the following, we discuss the distance measure, the summary statistics and the tuning parameter in detail in connection with each case study (See also the S1 File).

In SLInG, we settle for the probability density function of the standard normal distribution as our kernel function. We note that this is similar to the choice made in the synthetic likelihood approach [44]. Note, $\delta_{ABC}$ denotes an important tuning parameter that controls the overall level of sparsity. As $\delta_{ABC}$ is a normalizing factor for our ABC distance measure, it has a similar role as the error tolerance threshold used in rejection ABC methods (parameter values with $d(s_{sim},s_{obs})\gg \delta_{ABC}$ are likely to be rejected as the acceptance rate $\psi (d(s_{sim},s_{obs})/\delta_{ABC})\ll 1$). Alternatively, one can think of $\delta_{ABC}$ as a measure of our belief in the data as a smaller choice of $\delta_{ABC}$ could result in a closer fit to the data even if that requires a larger set of non-zero parameters in the model (See the Linear models section for this effect in practice).

SLInG uses Markov Chain Monte Carlo sampling based on a Metropolis-Hastings algorithm and Gibbs sampling building on the hierarchical ABC algorithm by Turner & Van Zandt [22]. In short, the algorithm samples the parameter space in *N* steps going through all *K* parameters in each step. The hierarchical nature of the problem is included by treating model parameters ($\theta_{k}$s) and hyper-parameters ($\epsilon_{k}$s) in separate loops. Firstly, hyper-parameters are sampled from the conditional probability given by Laplace prior based on parameter values from the previous sampling step. Then, in a second loop, new values for each $\theta_{k}$ are drawn independently from a proposal distribution *q*. Here, we use a normal distribution centred around the value of $\theta_{k}$ in the previous sampling step and with a variance of $\sigma_{s}^{2}$. Like $\delta_{ABC}$, the user must choose $\sigma_{s}^{2}$. However, the chosen value of $\sigma_{s}^{2}$ acts as an initial guess for a suitable step size. During the inference process, $\sigma_{s}^{2}$ is constantly updated based on the variance of the samples in the Markov chain, i.e., unique values for $\sigma_{s}^{2}$ are automatically set for each parameter. In this sense $\sigma_{s}$ plays the role of a learning rate for the proposal distribution: it controls how far the chain moves in a single Gibbs step, but in contrast to a fixed learning rate it is set adaptively from the running variance of the sampled $\theta_{k}$ values rather than specified by the user. Fig 1 offers an overall schematic overview of our approach, and the algorithm is also summarised in supporting information Section A in S1 File.

### Linear models

We treat the linear model as a validation case study: the diabetes data set of Efron et al. [25] is a standard benchmark in the sparse regression literature, used in the original Bayesian LASSO paper of Park and Casella [18], and it provides a setting in which we can verify that SLInG recovers the expected predictors before applying it to more complex problems. The progression of case studies in the paper, from this linear model through non-linear ODEs in the Adaptive protein signalling networks section to stochastic models in the Stochastic regulatory genetic networks section, allows us to demonstrate the capabilities of the algorithm systematically.

The diabetes data set by Efron et al. [25] is often used to demonstrate the performance of sparse sampling algorithms. The data set contains ten variables for 442 patients, and the task is to identify parameters that are predictive of disease progression (see also supporting information Section B.2 in S1 File). The model assumed to describe the data set is a simple linear model cf. [18].

To solve the task using SLInG, we employ the following distance measure:


$$\begin{array}{c} d=\frac{RMSD}{IQR}=\frac{1}{Q_{3}-Q_{1}}\sqrt{\frac{\sum_{j}^{J}(x_{j}^{sim}-x_{j}^{obs}{)}^{2}}{J}}, \end{array}$$ (2)

where $x_{j}^{obs}$ denotes the observed values of the response variable for the *J* patients and *Q*_1_ and *Q*_3_ denote the corresponding first and third quartiles (thus their difference is the interquartile range denoted as IQR). Here, $x_{j}^{obs}$ enters the root mean square distance (RMSD) together with the corresponding prediction ($x_{j}^{sim}$) of the linear model; the model contains no intercept since both the features and the response variable are centred around their corresponding mean.

**Fig 2:**
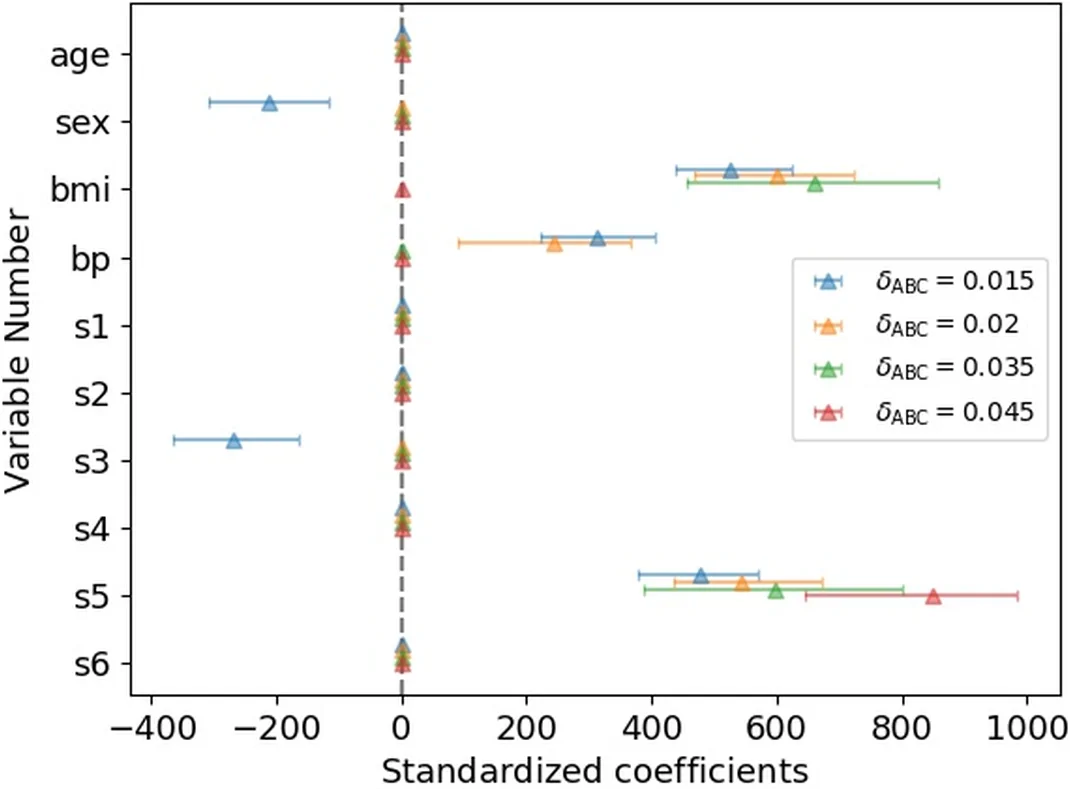
Parameter values inferred for the diabetes data set of Efron et al. [25] for different values of the overall sparsity parameter $\delta_{ABC}$. The plot shows the median (triangle) of each of the 10 parameters found by our sparse sampling algorithm. The error bars signify the corresponding 95 percent confidence intervals. Each of the Markov chains that underlie the plot contains 8,500 samples after excluding a burn-in phase, in which $\delta_{ABC}$ is iteratively adjusted to the requested value. The constrained parameters relate to patient attributes: age, sex, body mass index (bmi), average blood pressure (bp), total serum cholesterol (s1), low-density lipoproteins (s2), high-density lipoproteins (s3), total cholesterol/HDL (s4), serum triglycerides level (s5), and blood sugar level(s6).

The results of our fit to the data are summarised in Fig 2. As expected, higher values of $\delta_{ABC}$ lead to sparser solutions. At intermediate values of $\delta_{ABC}$ (0.02 and 0.035), we find that important predictors of disease progression are BMI and two of the six serum measurements, including glucose level, while lower values of $\delta_{ABC}$ indicate that other serum measurements and sex might play a role. This result is consistent with the literature, e.g., [18]. The order in which predictors are retained as $\delta_{ABC}$ decreases provides a natural ranking of their relative importance: BMI and the blood-glucose-related serum measurement are selected first and remain non-zero across the widest range of $\delta_{ABC}$, followed by sex and the remaining serum measurements at tighter tolerances. This ranking reproduces the prioritisation reported in the diabetes literature [18,25], where body mass index and glucose are the most consistently identified drivers of progression and sex enters as a secondary modulator.

**Fig 3:**
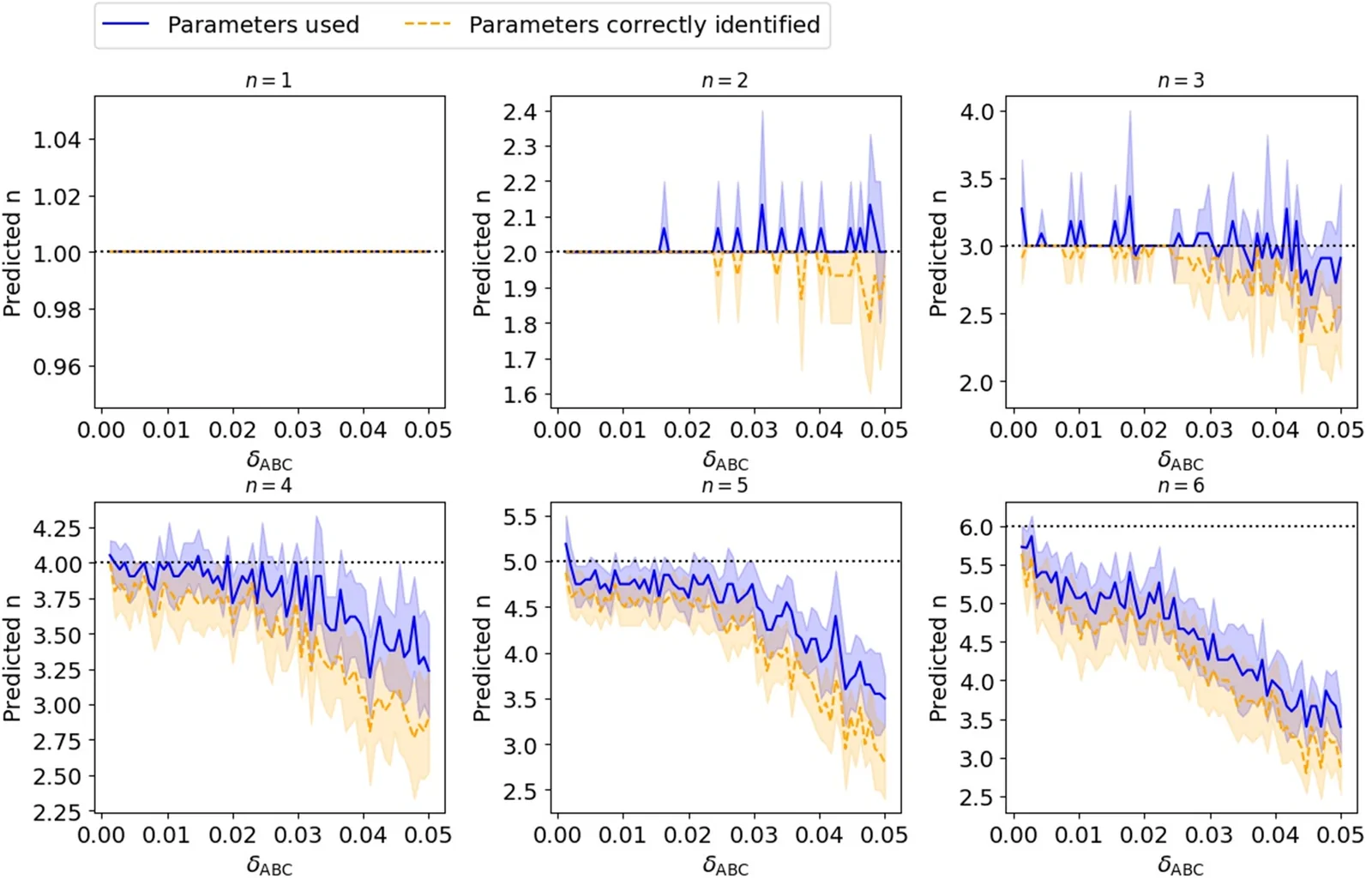
Parameters identified by SLInG as a function of $\delta_{ABC}$. We fitted 100 synthetic data sets describing a simple linear system with 10 free parameters, varying the number of non-zero parameters (*n*) between 1 and 6 and the associated parameter values within two orders of magnitude. In each case, we repeated the inference for 66 values of $\delta_{ABC}$. The plot shows the mean number of non-zero parameters needed by the algorithm (blue) for each value of *n* and highlights the correctly identified parameters (orange) together with the corresponding 95 percent confidence intervals. The plot is based on the best-fitting models from each Markov chain.

To further quantify the performance of the Gibbs sampler, we used the data set from Efron et al. to construct synthetic data sets, i.e., we substituted the response variable with model predictions with known ground truth parameters and added noise (for details, see supporting information Section B.2 in S1 File). Since we know the parameter values that were used to create the synthetic data sets, this approach allows us to perform self-consistent tests. By doing so, we can check whether the Gibbs sampler is able to accurately reproduce the ground truth, i.e., the parameter values of the synthetic data. To map the impact of $\delta_{ABC}$, we repeat the inference for each of the synthetic data sets using different values of $\delta_{ABC}$. The results are summarised in Fig 3.

At low values of $\delta_{ABC}$, SLInG consistently recovers the ground truth, pinpointing the parameters that take non-zero values and discarding the rest. As $\delta_{ABC}$ increases, the sampler selects models that are sparser than the ground truth. However, while the sampler points to solutions that are sparser than the model that generated the data, we note that the sampler points to parameters that are still a subset of the correct ones, i.e., the sampler picks up on the correct system dynamics. We also note that this behaviour is consistent with the results shown in Fig 2. When simpler models are inferred for larger values of $\delta_{ABC}$, the parameters associated with variables that have the largest explainability are kept, as shown in Fig S1 in the S1 File. This finding has desirable implications. Namely, that noise or sparsity in the data will result in a sparse approximation of the true model since noise and sparse data inevitably result in a larger distance between the model and the data. Overall, we find that the performance of SLInG is not too sensitive to the value of our sparsity tuning parameter $\delta_{ABC}$, as long as this parameter is not too large to enforce sparsity priors to overwhelm the signal from data, leading to overly sparse solutions that put too many parameters to zero. Ultimately, the appropriate level of sparsity obtained is related to the level of confidence in the summary statistics. A less sparse model could fit the data closely, but this should be avoided if it represents over-fitting (beyond the confidence we have over the data).

**Fig 4:**
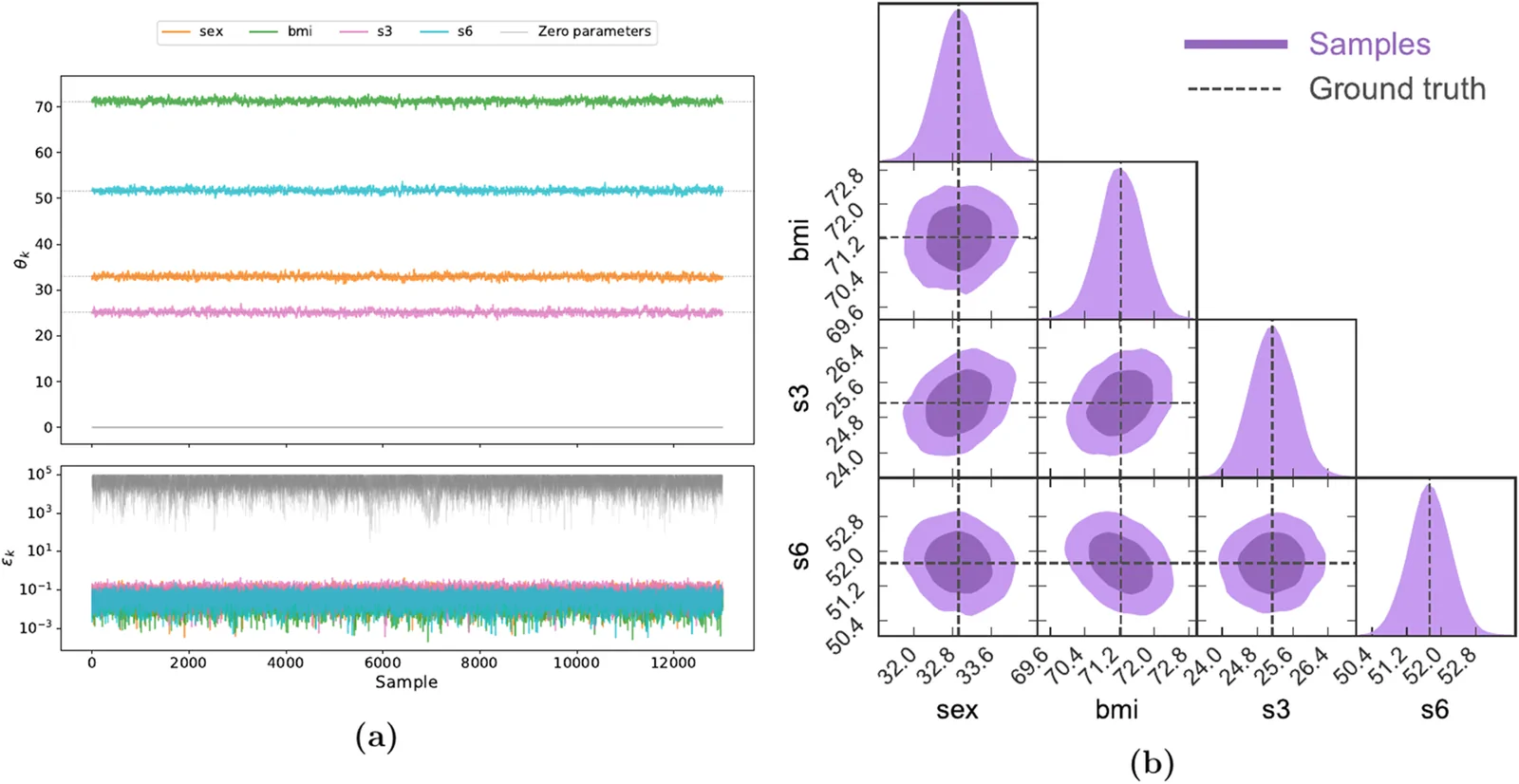
Markov chain for a single synthetic data set with four non-zero parameters. (a) The top plot shows the sampled parameter values ($\theta_{k}$) at each iteration, with dashed grey lines indicating the ground truth. The bottom plot shows the corresponding hyper-parameters ($\epsilon_{k}$) in the Laplace prior. We only include non-zero parameters in the legend while we plot all ten parameters and hyper-parameters across the 10,000 samples. (b) The resulting posterior distribution for the four non-zero parameters. All four parameters were correctly identified in this example. In both panels the four non-zero parameters are drawn in distinct colours and labelled in the legend, while the six parameters whose ground-truth value is zero are drawn in grey; the same colour/grey convention applies to the trajectories in panel (a) and to the posterior densities in panel (b).

Although we only include the best-fitting models in Fig 3, we are not limited to providing a point estimate. Indeed, this is one of the great advantages of Bayesian approaches, such as Monte Carlo algorithms: SLInG provides the posterior distributions for the obtained parameter set. We illustrate this for one of our synthetic data sets in Fig 4. Also, the figure highlights that the algorithm autonomously sets hyper-parameters (the $\epsilon_{k}$s) to be either very large (indicating that the corresponding parameter should be zero) or relatively small (suggesting the parameter of interest is set to have a non-zero value). The oscillations visible in the $\epsilon_{k}$ traces of Fig 4a are a feature of the Gibbs scheme rather than a sign of non-convergence. Each $\epsilon_{k}$ is resampled at every iteration from its conditional posterior given the current value of $\theta_{k}$; because $\theta_{k}$ itself fluctuates across the chain, the conditional posterior for $\epsilon_{k}$ shifts accordingly and produces the visible oscillations. The relevant convergence diagnostic is that the $\epsilon_{k}$ values for non-zero parameters remain consistently small while those for zero parameters remain consistently large, and this bimodal separation is clearly achieved in Fig 4a, confirming convergence in terms of the stationary distribution.

### Adaptive protein signalling networks

**Fig 5:**
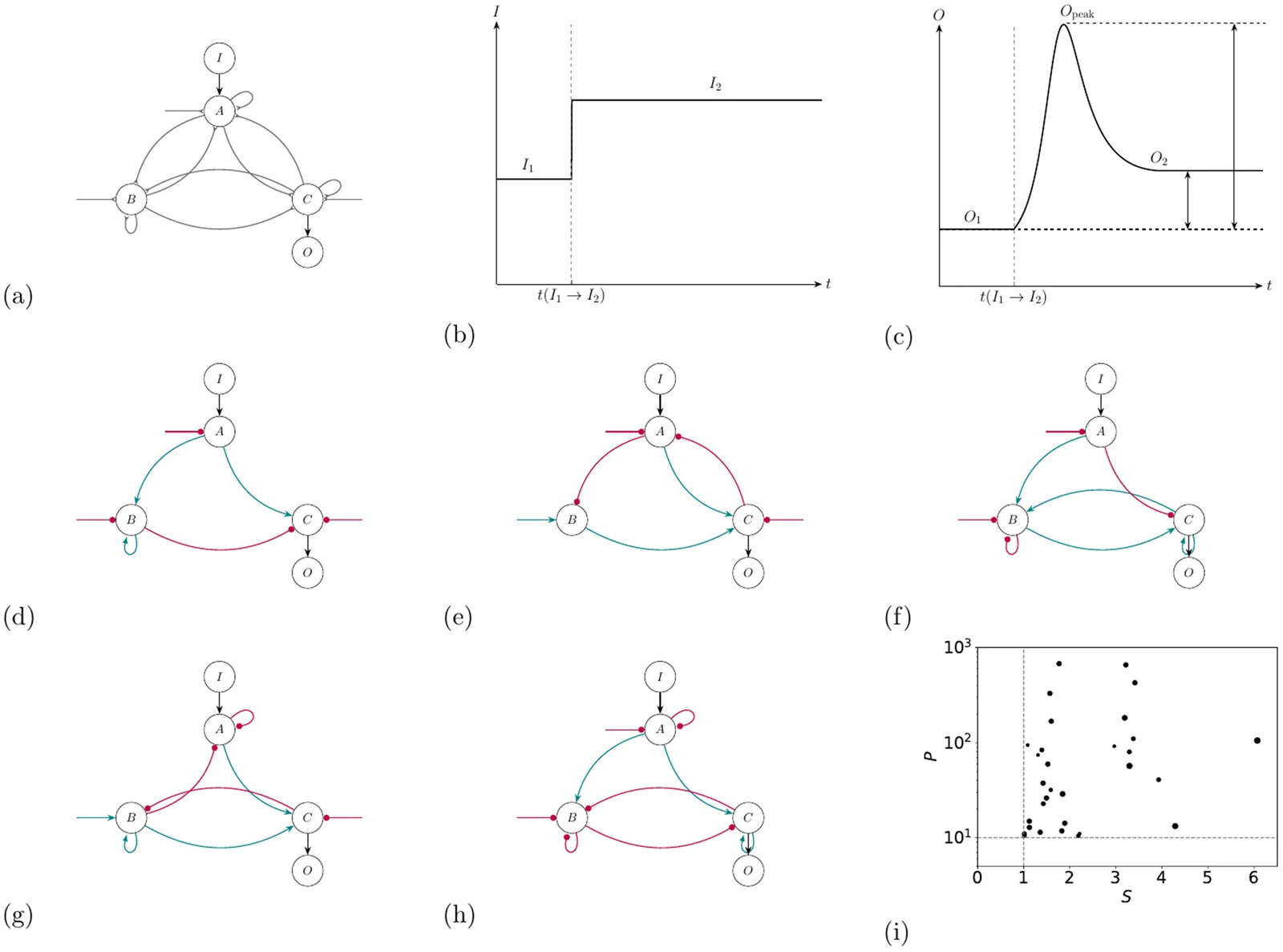
Summary figure for signalling networks exhibiting adaptation. (a) General protein network structure. Each network contains three proteins (A, B, C). (b) Change in the input (*I*). (c) Adaptive response in the output (*O*) to the change in the input. (d)-(h): Subsample of the adaptive networks found by the sampler. Red, blunt-headed arrows indicate repression, while cyan arrows indicate activation. Note, e.g., that (e) is an example of negative feedback, while (d) is an example of an incoherent feedforward loop. While we only show five networks here, our algorithm has successfully identified many more (cf. the supporting information). (i) *S* and *P* for all unique networks listed in the supporting information. The dashed line highlights the definition of adaptation: *S* > 1 and *P* > 10.

Adaptive protein signalling networks respond to changes in an input signal (*I*) with an output signal (*O*) (Fig 5a). Any change to input signal ($I_{1}\to I_{2}$) initially leads to a strong response in the output signal (Fig 5b and 5c). However, after some time, the network will adapt to the change in the input signal, and the output signal will return close to its pre-stimulus level (Fig 5c). This kind of response occurs in different types of sensory systems, such as chemical receptors in olfactory cells [26]: while a new smell will initially provoke a strong response, it will disappear into the background with time. The chemotaxis of bacteria provides other examples of adaptive systems [45].

Ma et al. [28] set out to identify adaptive biochemical network topologies with three proteins: A, B, and C. A is receptive to the input, while C transmits the output. The three proteins might be linked to each other (thus repressing or activating each other) or possess self-feedback loops. Moreover, each protein might be activated or deactivated by other enzymes, for example, through phosphorylation/dephosphorylation reactions ($E_{A}$, $E_{B}$, $E_{C}$). The supporting information summarises the ordinary differential equations (ODEs) describing such networks (cf. supporting information Section B.3 in S1 File). In their paper, Ma et al. thus address the question of which particular network topologies result in adaptive behaviour. To answer this question, they define adaptation based on two quantities that they denote as sensitivity and precision. The sensitivity (*S*) encapsulates the initial reaction of the network:


$$\begin{array}{c} S=\left|\frac{(O_{peak}-O_{1})/O_{1}}{(I_{2}-I_{1})/I_{1}}\right|, \end{array}$$ (3)

where *O*_1_ and $O_{peak}$ denote the initial and extreme value in the output signal. *I*_1_ and *I*_2_ are the initial and final input signals. The precision (*P*) quantifies the successful return to the prestimulus level:


$$\begin{array}{c} P={\left|\frac{(O_{2}-O_{1})/O_{1}}{(I_{2}-I_{1})/I_{1}}\right|}^{-1}, \end{array}$$ (4)

where *O*_2_ denotes the final output signal. Networks are said to be adaptive when both *S* > 1 and *P* > 10.

Ma et al. identify adaptive networks through a brute-force search of all network topologies in their parameter space. To address this rather abstract task with SLInG, we use the following distance measure:


$$\begin{array}{c} d=1-\frac{S^{n}P^{n}}{\left(S^{n}+k_{s}^{n})(P^{n}+k_{p}^{n}\right)} \end{array}$$ (5)

that is, a non-linear, increasing function of sensitivity and precision. We set *n* = 2, $k_{s}=1$, and $k_{p}=10$. Like Ma et al., we set *I*_1_ = 0.5 and *I*_2_ = 0.6. This choice for the distance measures allows for selecting networks with a qualitative constraint on their behaviour rather than fitting a specific data set. The priors of the model parameters and details of the set-up are described in supporting information Section B.3 and supporting information Section B.4 in S1 File.

Based on one hundred independent Markov chains, we identified 31 unique topologies showing adaptation. Fig 5 shows five examples. All networks are included in the supporting information (Figs S2-S3 in S1 File). The 31 successful topologies were all found when choosing low values of $\delta_{ABC}$. This behaviour was to be expected as higher values of $\delta_{ABC}$ imply that the imposed sparsity requirements pull the sampler away from the relevant region of the parameter space.

Ma et al. suggest that two topologies lie at the heart of any adaptive network: a negative feedback loop with a buffer node (node B in Fig 5) and an incoherent feedforward loop with a proportioner (also node B in Fig 5). Indeed, all the networks identified by SLInG fall into these categories.

We note that an exhaustive enumeration by Ma et al. [28] returned 395 three-node topologies capable of adaptation, whereas SLInG recovers 31 of them. This difference reflects the difference between the two search strategies rather than a deficiency of SLInG: the exhaustive procedure of Ma et al. sweeps every topology and every node of a dense parameter grid, while SLInG performs a sparsity-biased stochastic search that preferentially returns the simplest networks consistent with adaptation and accumulates samples in regions of high posterior probability. Importantly, the 31 topologies found by SLInG all sit within Ma et al.’s two core adaptive motif classes (negative feedback with a buffer node and incoherent feedforward with a proportioner) and therefore represent the parsimonious core of the 395. A SLInG user seeking a broader coverage can soften the sparsity prior or run additional chains; the trade-off is between coverage of the topology space and the preference for parsimonious solutions that motivates SLInG in the first place. In Fig 5, panel (e) is an example of a negative feedback loop: A activates C, but C represses A. Meanwhile, in Fig 5, panel (d) is an example of an incoherent feedforward loop: A activates C, but through its activation of B, A also represses C. In addition, several of the networks are found to use a combination of these strategies.

In this Section, our aim is to identify topologies that allow for adaptation. Our research question is thus about the existence of such topologies: We are interested in whether we can construct sparse networks that exhibit adaptation but not in the precise parameter values. Thus, in contrast to the analysis presented in the Linear models section, we do not aim to constrain the parameter values. Since we are not making any statements about the posterior distribution of the parameter values, we do not need to ensure convergence of the Markov chain. However, a closer look at the Markov chain might still give additional insight into the identified signalling network. As noted by Ma et al., one might ask whether any given network topology robustly results in adaptation for a broad range of parameter values or whether some parameters need to be fine-tuned to ensure adaptation. Each of the networks might thus correspond to either a broad or a narrow peak in the posterior probability landscape. Indeed, one of the advantages of using a Monte Carlo approach is that one can detect fine-tuned topologies that would be overlooked in a coarse grid search of the parameter space. Just like in the case of a grid search, we can make qualitative statements on whether the identified topologies robustly lead to adaptation if we have obtained enough independent samples, even if the Markov chain has not converged to the posterior. We illustrate this point in Figs S4 and S5 in the S1 File. The network topology illustrated in Fig S4 in S1 File is more robust than the one topology illustrated in Fig S5 in S1 File as evident by the wider range of parameter values resulting in adaptation.

Importantly, our case study illustrates that SLInG can go beyond the simple linear models exemplified in the Linear models section. As discussed in the supporting information, the ODEs used to model this system are relatively high-dimensional, including 26 free parameters, and are also non-linear.

### Stochastic regulatory genetic networks

Having demonstrated that our sparse sampling algorithm can be successfully applied to linear and non-linear models in the Linear models and Adaptive protein signalling networks sections, we proceed to discuss a stochastic example: reverse engineering gene regulatory networks from single-cell transcription data.

Before describing the case study, we note the rationale behind two design choices. We work with four-gene networks because this size balances biological relevance with tractability: small regulatory modules of three to five genes appear repeatedly in development, signalling and metabolism, while the number of possible directed, signed topologies grows combinatorially with the number of genes and quickly becomes difficult to either simulate exhaustively or visualise. Four genes are enough to give non-trivial covariance structure across pairs of genes and small enough to benchmark against a known ground truth. We also exclude self-activation and self-repression from the inference as a simplifying assumption, so that the sparsity prior acts purely on inter-gene links. This is not a methodological limitation of SLInG, which can in principle handle self-regulatory edges by extending the set of free parameters; we focus on inter-gene interactions because these are the edges of primary interest when reconstructing a regulatory topology from population-level expression data and because self-loops are only weakly identifiable from snapshot covariances.

The topologies of gene regulatory networks can be inferred from single-cell gene expression data, e.g., single-cell RNA-sequencing data. However, this inference task is challenging because gene expression is known to be affected by intrinsic noise in biochemical reactions [46]. Intrinsic noise can lead to significant variability in gene expression levels, even for genes that are regulated by the same factors. This variability can obscure the true underlying regulatory relationships and make it challenging to distinguish real regulatory interactions from noise. In addition, typically, we do not have access to single-cell time-course data and/or perturbation data, so we are limited to snap-shot data.

**Fig 6:**
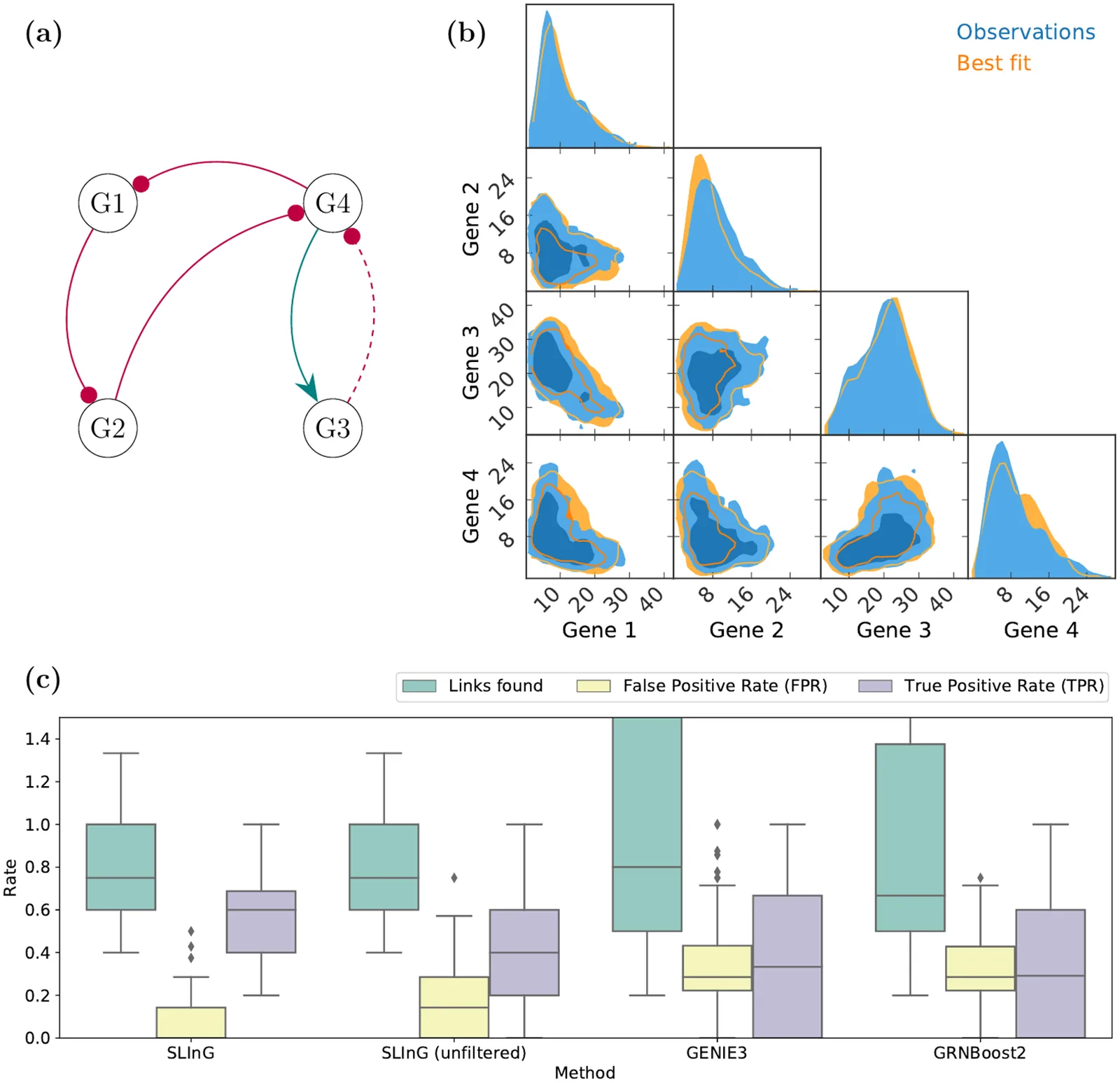
Performance summary for stochastic models of gene regulatory networks. (a) The network that underlies the particular synthetic data. The Gibbs sampler correctly identified four out of the five links. The link that was not found is indicated with a dashed line. (b) Examples of probability density contours for predicted and observed gene expression across 1,000 cells. (c) We compare our sparse Gibbs sampling algorithm (SLInG) with two established gene network inference tools from the literature, GENIE3 and GRNBoost2. We include the results for SLInG for the 56 simulations that passed our quality check, as well as for all 100 networks. The plot includes the number of links that the algorithms settle for as a fraction of the true number of links. Moreover, we list the true and false positive rates, summarising how many links were correctly identified and how many spurious links were claimed to be found. The true and false positive rates are defined as *TPR* = *TP*/*P* and *FPR* = *FP*/*N*, respectively. *TP* and *FP* denote the total number of true and false positives, respectively, while *P* and *N* denote the numbers of positives and negatives in the data. Thus, while *TP* and *FP* stem from a comparison between the model predictions and the ground truth, *P* and *N* are based on the ground truth alone.

To illustrate the performance of SLInG for model-based inference of genetic networks from data, we choose to work with synthetic data since this allows us to perform a self-consistent test and hence to benchmark SLInG (see also the Comparison to other algorithms section). Here, we focus on gene regulatory networks that contain four genes. The links between these genes might lead to either repression or activation of gene expression. We do not consider self-activation or self-repression. Gene-regulatory networks are often simulated using the Gillespie algorithm that respects the discreteness of the molecules [47]. However, in this paper, we employ a stochastic differential equation (SDE) approach due to computational efficiency (see supporting information Section B.5 in S1 File). For this purpose, we employ a recently published library for biochemical modelling by Loman et al. [48] to produce synthetic data and to perform a self-consistent test, i.e., SLInG draws on the same library to infer the model parameters that underlie the synthetic data. The output of the model is a distribution of gene expression counts across single cells, capturing gene-gene correlations that contain information with regard to the underlying network (cf. Fig 6c below). The output of simulations then closely mimics real-world experiments with cell populations. Here, we focus on the difficult case of working with only snap-shot data and do not include time-course data and/or perturbation data.

To fit the data, we use the first two moments of gene expression as summary statistics [49] and employ the following Euclidean distance:


$$\begin{array}{c} d=\sqrt{\frac{1}{N_{c}}\left(\sum_{j=1}^{4}\frac{({\bar{x}}_{j}^{sim}-{\bar{x}}_{j}^{obs}{)}^{2}}{\sigma_{j}^{2}}+\sum_{g=1}^{16}\frac{(C_{g}^{sim}-C_{g}^{obs}{)}^{2}}{\sigma_{g}^{2}}\right)}, \end{array}$$ (6)

where ${\bar{x}}_{j}^{obs}$ and ${\bar{x}}_{j}^{sim}$ denote the observed and predicted mean expression of gene *j*, respectively. The standard deviation ($\sigma_{j}$) of the observed mean expression is determined by bootstrapping. $C_{g}^{sim}$ and $C_{g}^{obs}$ denote the 16 elements of the covariance matrices of the obtained distribution of gene expressions for the model predictions and the synthetic observations, respectively. The standard deviations ($\sigma_{g}$) of the element of the covariance matrix of the observations are found by bootstrapping. Finally, $N_{c}$ denotes the number of cells in the experiment. Here, the cell population is assumed to contain 1,000 cells. We created one hundred synthetic data sets based on random 4-node gene-regulatory networks. For further details, we refer the reader to supporting information Section B.6 in S1 File.

In the Linear models and Adaptive protein signalling networks sections, we show that higher values of $\delta_{ABC}$ yield sparser solutions and that we fully employ the information in the data when setting $\delta_{ABC}$ to a very low value. In the present case, this approach fails. Since the model is stochastic, SLInG is prone to get stuck if it becomes too sensitive to changes in the distance measure. After all, the distance measure will vary between two samples with the *same* parameter values due to stochasticity and the finite sample size (1000 cells). Indeed, Monte Carlo methods are known to struggle with stochastic simulations, e.g., [50–52]. However, if we increase the value of $\delta_{ABC}$, the sampler becomes less sensitive to the stochasticity of the simulations and hence becomes effective for the inference based on stochastic simulations. Just by varying $\delta_{ABC}$, we can thus overcome an obstacle that would otherwise severely stifle our algorithm. For further details on the synthetic data and priors, we refer the reader to supporting information Section B.6 in S1 File.

We fitted each synthetic data set using SLInG and subsequently assessed the quality of each fit (cf. supporting information Section B.6 in S1 File). If we were faced with a low-quality fit when applying the algorithm to real-world data, we could try to improve the fit by, e.g., adjusting initial conditions. Here, however, we discard low-quality fits and note that the algorithm passed our quality check out of the box in 56 percent of the cases.

On average, the number of non-zero links in the best-fitting models corresponds to 79 percent of the number of links used to create the synthetic data set in question. Note that it is expected that the algorithm will not find all links due to the requirement of sparsity, our conservative cut-off in the link strength, and the expected trade-off between the link strengths, the values taken by base expression rates and model simplicity (supporting information Section B.5 in S1 File). Notably, 80 percent of the predicted links correctly identify a link between genes in the synthetic data *and* attribute the correct sign to the interaction. That is, we strictly distinguish between repressive and activating links. In other words, 80 percent of the predicted links are really present in the data. Combining these two numbers, we achieve a true positive rate of 60 percent, i.e., on average, the algorithm correctly identified 60 percent of all the links present in the data. Moreover, in every case, at least one link was correctly identified. This is a very promising result. Indeed, as discussed in the Comparison to other algorithms section and as shown in Fig 6c, our sampler outperforms algorithms explicitly designed to identify links in gene regulatory networks.

To further illustrate the performance of SLInG, we include an example of a fit to the synthetic gene expression data in Fig 6b.

### Comparison to other algorithms

We compare our method with two of the most commonly used and high-performing gene regulatory network inference algorithms: GENIE3 and GRNBoost2 see [40,53–55] as well as supporting information Section B.7 in S1 File for further details. Both algorithms are well-established in the field. By combining their output with the ‘ppcor’ package in R to compute the directionality of the interactions [56], we can produce estimates of signed, directed networks that we can compare with SLInG.

As can be seen from Fig 6c, both GENIE3 and GRNBoost2 often overestimate the true number of links, while the median number of links obtained with both algorithms is similar to that obtained with SLInG. Moreover, GENIE3 and GRNBoost2 lead to a significantly higher number of false positives and a somewhat lower number of true positives than SLInG. Even if we were to fix the number of links to the ground truth, this statement would still hold true. The high number of false positives is thus not solely an artefact that stems from overestimating the true number of links. This behaviour is a direct expression of the precision/sensitivity (equivalently precision/recall) trade-off inherent in tree-ensemble methods: by ranking a large number of candidate interactions without a built-in shrinkage step, GENIE3 and GRNBoost2 achieve reasonable sensitivity at the cost of precision, while SLInG’s sparsity prior shifts the operating point towards higher precision by actively discarding weakly supported links.

We note that SLInG has an advantage that further boosts its scores in our comparison: The output of SLInG allows us to compare the predicted network structure with the data directly. We can hence impose a quality check based on which we can discard bad fits. GENIE3 and GRNBoost2 allow for no such filtering. If we abandon the quality check offered by SLInG, its median true positive rate drops from 0.6 to 0.4, lying closer to that of both GENIE3 and GRNBoost2 (cf. Fig 6). This being said, one of the appeals of using SLInG lies in its transparency: Because SLInG draws on generative models, we can directly compare the model output to the data in posterior predictive tests or through the goodness of fit. One wouldn’t abandon such quality checks when dealing with real-world data.

### Benchmarking on real mouse embryonic stem cell scRNA-seq (BEELINE)

To complement the self-consistent synthetic benchmark above, we evaluated SLInG on real single-cell RNA-sequencing data from the mouse embryonic stem cell (mESC) dataset distributed with BEELINE [57]. Starting from the BEELINE mESC expression matrix we pooled all five available time points (00h, 12h, 24h, 48h, 72h) to obtain *n* = 421 cells and assembled a candidate pool by taking the union of all 1078 expressed mouse TFs (from the BEELINE distribution) with the top-500 most variable genes, giving 1516 unique candidate genes (62 of the top-500 are themselves TFs and so contribute to both sets). We then sampled 25 random eight-gene subnetworks rooted at transcription factors with at least one experimentally supported loss-of-function/gain-of-function (lofgof) target in the pool, filling each subnetwork with seven other genes from the pool. Reference edges per subnetwork are the BEELINE lofgof entries whose endpoints both fall in the eight-gene subset (1–3 edges per subnetwork). GENIE3 and GRNBoost2 are applied to the same eight-gene expression matrices as direct baselines, matching the scope of Fig 6c on real data.

Two methodological adjustments were required to move from the self-consistent CLE-synthetic setting to real data. First, the simulator’s per-gene parameters (steady-state target mean, technical dropout rate, and intrinsic-noise scale) were calibrated per subnetwork to match the mean, zero fraction, and non-zero variance of the observed log-normalised expression respectively; the underlying birth/death kinetic constants are unchanged from the published pipeline. This calibration is a single-parameter-per-gene update and is necessary for the ABC distance to probe covariance structure rather than trivial mean mismatch. Second, we summarise each interaction coefficient’s posterior by its z-statistic, ${SNR}_{k}=|mean(\theta_{k})|/std(\theta_{k})$ (the absolute value of the signed posterior mean normalised by posterior standard deviation) rather than by $\left|median(\theta_{k})\right|$ as used for the self-consistent benchmark. The z-statistic is the natural Bayesian summary of a posterior on an effect coefficient; we corroborated this approach on 20 self-consistent CLE-synthetic 4-gene networks and found that SNR is statistically equivalent to the median-based rule on that benchmark (Δ mean AUROC =−0.02, 95% bootstrap CI [−0.13,+0.09]), while it is substantially better on real scRNA-seq data where non-regulatory edges between co-expressed genes drive large but uncertain posteriors that magnitude-only summaries misrank (see S1 File).

Three ranking metrics are reported. AUROC is the area under the receiver operating characteristic curve over all G(G−1) directed edges per subnetwork, treating the BEELINE lofgof entries as positives. Early precision at K (EP@K) is the precision among the top-K predictions per subnetwork, where K equals the number of reference edges in that subnetwork; it tests whether each method’s highest-confidence calls coincide with the reference network. Recall@10 is the fraction of reference edges recovered within each subnetwork’s top-10 predictions; it tests whether reference edges are reachable within a fixed shortlist size that does not scale with subnetwork sparsity. All three are averaged across the 25 subnetworks. EP@K and recall@10 sidestep the choice of a global score threshold by working with each subnetwork’s own ranking, which matters when comparing a posterior probability (SLInG) against importance scores (GENIE3, GRNBoost2) on different scales.

Table 1 summarises the performance of each method. On the 25 subnetworks, SLInG achieves mean AUROC 0.61 (95% bootstrap CI [0.52, 0.71], 10 000 resamples), outperforming GENIE3 (0.35, [0.25, 0.46]) and GRNBoost2 (0.37, [0.27, 0.48]). Paired-bootstrap comparisons across subnetworks give advantages of SLInG over GENIE3 of +0.26 (95% CI [+0.12, + 0.40], 20/25 subnet wins) and over GRNBoost2 of +0.24 (95% CI [+0.10, + 0.39], 19/25 wins); both confidence intervals comfortably exclude zero. The other two metrics tell a consistent story (Table 1): SLInG dominates both baselines on EP@K (0.16 vs 0.00 / 0.02) and on recall@10 (0.30 vs 0.14 / 0.08), well above the random expectations of 0.026 and 0.179 respectively, so the ranking advantage is not an artefact of the AUROC summary. The margin by which SLInG outperforms GENIE3 and GRNBoost2 on real mESC scRNA-seq is of comparable scale to the margin reported in Fig 6c on CLE-synthetic data, demonstrating that the original conclusion is not an artefact of the self-consistent evaluation.

**Table 1:** Mean AUROC, EP@K and recall@10 across 25 randomly sampled eight-gene TF-rooted subnetworks of the BEELINE mESC scRNA-seq dataset, scored against BEELINE’s lofgof reference edges. EP@K is precision among the top-*K* predictions per subnetwork (where *K* is the number of reference edges in that subnetwork); recall@10 is the fraction of reference edges recovered in each subnetwork’s top-10 predictions. Random expectation under uniform ranking is $\bar{K}/[G(G-1)]=0.026$ for EP@K and 10/[G(G−1)]=0.179 for recall@10 (with *G* = 8).

Method	AUROC	EP@K	Recall@10
SLInG (z-statistic)	0.610	0.160	0.300
GENIE3	0.350	0.000	0.140
GRNBoost2	0.368	0.020	0.080
Random expectation	0.500	0.026	0.179

Four of the twenty-five subnetworks (those rooted at Esx1, Zfp42, Sox2, and Nanog) yielded an AUROC of exactly 1.000 for SLInG; these are core mESC pluripotency regulators with well-characterised downstream targets, so the result is biologically coherent. We note that lofgof is a reference rather than a gold standard: single-gene perturbation data misses redundantly regulated edges and can include indirect cascade effects, so AUROC is conservative. However, this limitation affects all three methods equally, so the paired comparisons remain informative.

## Discussion

Inference of genetic and signalling networks is a fundamental and challenging task in systems biology. Some of the current methods rely on obtaining correlation networks and are not mechanistic. Using ODEs or stochastic models of biochemical networks is challenging, as the network inference task would be equivalent to large-scale model selection among many competing network topologies. In this paper, we cast the problem as model/equation discovery using Bayesian sparse inference and demonstrate that our implementation reliably pinpoints the correct non-zero parameters relating to the regulatory links existing in the network in self-consistent tests. Our approach relies on a sparsity-inducing Gibbs ABC algorithm that we call SLInG. It is applicable to any simulation-based inference task, including non-linear, stochastic or agent-based models see [58] for an application of SLInG to agent-based models within the realm of environmental conservation studies. Furthermore, the algorithm successfully adjusts the level of sparsity in a data-dependent fashion for each parameter individually, resulting in models with the appropriate level of complexity.

In the Linear models section, we address a well-studied linear model for the occurrence of diabetes among patients. We demonstrate that SLInG correctly identifies the non-zero parameters when faced with synthetic data and yields results that are consistent with the literature when dealing with real-world data. We show that our Bayesian algorithm yields the joint posterior distribution of all parameters, quantifying the uncertainty of the model predictions see also [4,18,59] for other Bayesian algorithms for equation discovery that provide posteriors. In contrast, many sparse sampling schemes only provide point estimates, e.g., classical LASSO or some state-of-the-art approaches within equation discovery, e.g., [1,3].

In the Adaptive protein signalling networks section, we successfully identify signalling networks that exhibit a particular biochemical function: adaptation. Following Ma et al. [28], who performed an exhaustive grid search to identify such systems, we consider signalling networks with three genes. Such systems are well-studied, allowing for a comparison to the literature. As our approach can be used with any distance function between data and model predictions, we can use a distance function that optimises qualitative aspects of the response to represent an adaptive response in this case. We thus see that SLInG consistently points to signalling network topologies that show the expected features: negative feedback loops with a buffer node and incoherent feedforward loops with a proportioner. Moreover, based on the samples in the Markov chain, we can directly compare the robustness of different network topologies.

An exhaustive search of all network topologies and a grid parameter search in each case, such as the one performed by Ma et al., fully covers a region of the parameter space at a predetermined resolution, which might become computationally insurmountable as the number of parameters increases [28,31,32]. In contrast, a Monte Carlo algorithm, such as SLInG, accumulates more samples from regions with high posterior probability, efficiently combining scouting for topologies and inferring parameters that meet given selection criteria. It would thus be computationally feasible to extend the search for adaptive topologies beyond signalling networks with three genes using SLInG, while the same would not hold true for a grid search of the parameter space. We note that our formulation of the problem is related to a recently proposed approach by Miltra et al. [60] in that it incorporates qualitative information into the inference of systems biology models [60].

In this connection, we note that while our Gibbs sampling algorithm scales relatively well when dealing with models with a higher number of parameters, it is inevitable that we will face the curse of dimensionality for vastly more extensive networks. Our search for 3 node adaptive models used models with 26 parameters. As the number of parameters increases, the time for a single Gibbs sampling run increases linearly with the number of parameters, but it will also take longer for a single chain to fully explore the posterior distribution depending on problem specific complexity of the posterior. Using multiple runs on a parallel machine can speed up a full exploration of the parameter space. Thus, besides the curse of dimensionality, another problem is the computational cost of the increased number of simulations required. However, in the last few years, a promising new line of research based on different machine learning approaches has emerged that can accelerate simulation-based inference [52,61–63], and some of these approaches can be applied in tandem with SLInG to overcome the stumbling block imposed by computational costs. An interesting future direction would be adopting these methods for model discovery and sparse Bayesian inference (see, e.g., [64]).

Moreover, by looking for adaptive signalling networks, we demonstrate that SLInG can successfully handle parameters that do not simply enter as coefficients in a linear combination of source terms. In contrast, some classic methods in equation discovery, such as sparse identification of non-linear dynamics (SINDy), are designed to constrain a matrix of linear combination coefficients Ξ, e.g., [1,3,4,59]. In other words, while the resulting dynamics are thus non-linear, the equations explored by SINDy must take the form ${\dot{𝐱}}^{T}=\Theta (𝐱)\Xi$, where 𝐱 denotes the state of the system and Θ(𝐱) denotes a matrix of library terms that may enter the ODEs that describe the systems dynamics. We note that there have also been some recent advancements in SINDy methods that extend the original framework’s capabilities. While the standard SINDy algorithm identifies systems as a linear combination of predefined basis functions, SINDy-PI [65] allows for the recovery of implicit dynamics and rational nonlinearities, expanding the scope of models it can infer. Moreover, Implicit-SINDy [66] has been introduced to recover implicit ordinary differential equations, while SINDy with Control (SINDYc) [67] incorporates control inputs into the framework, making it suitable for systems influenced by external factors. Additionally, Weak SINDy [68] employs integral formulations to better manage noisy data, enhancing the robustness of the method in practical applications. These advancements provide powerful extensions to the original SINDy framework, allowing for more flexible and accurate model discovery, which complements the approach proposed in this work. Specifically, in the context of learning deterministic reaction networks based on mass action kinetics, Reactive SINDy [69] was developed. More recently, Jiang et al. [70] extended Reactive SINDy using a Bayesian framework with a regularised horseshoe prior for dynamic biochemical networks inference, improving model inference from sparse observations as the method does not rely on estimation of derivatives. Finally, there are other Bayesian approaches that have been proposed for Bayesian MCMC search in the space of closed-form mathematical models, such as the so-called “machine scientist” that can be used for inference of ODEs and is more flexible in terms of non-linear combination of equations [71]. However, most of these methods cannot be applied to fully simulation-based problems such as stochastic models and used with arbitrary ABC distance measures as demonstrated in our examples in this study.

As discussed above, some authors have performed exhaustive searches over the space of all network topologies to address this kind of non-linear model discovery problem in the context of systems biology. For example, Beik et al. propose a novel Bayesian multi-model approach to search the space of mechanistic hypotheses that are consistent with data [72]. We also note that mixed-integer optimisation methods have also been applied successfully to search for network topologies with desired functional properties in the context of network design in synthetic biology, as demonstrated by Otero-Muras and Banga (2017) [73]. These approaches can scale well to larger networks, however as they are optimisation based, they do not have the advantages of Bayesian methods. Other authors have used methods based on genetic algorithms and *in silico* evolution [33–35]. These methods draw on a library of genotypes that represent specific networks and their parameter sets. The algorithms use rounds of mutations and selection to find fitter networks. In principle, these methods could explore an unlimited level of complexity to obtain solutions given an appropriate set-up for the allowed mutations in the system. In contrast, in our approach, we specify the maximum level of complexity at the outset and look for the simplest sub-networks that perform the desired task or fit the available data. Our case study of adaptive systems suggests that SLInG can provide a more versatile and efficient option to existing *in silico* evolution approaches.

To showcase the application of SLInG to a stochastic model, we reverse engineer the topology of gene regulatory networks based on gene expression data in the Stochastic regulatory genetic networks section. Like the identification of signalling networks with particular functions, inference of genetic networks from snap-shot gene expression data is an important open problem in computational systems biology [38]. Many algorithms have thus been proposed to address this task and have, over the years, been applied to bulk, single-cell, temporal and perturbation data. Many of the existing methods are not based on mechanistic models, and they merely try to reconstruct a correlation network. We demonstrate that SLInG shows good performance when compared to some of the leading correlation-based algorithms (GENIE3 and GRNBoost2) that are specifically tailored to reverse engineer gene regulatory networks. Moreover, we note that we perform the reverse engineering of signalling networks based on stochastic models (SDEs). While some Monte Carlo algorithms struggle to explore the parameter space when faced with stochastic models, we show that our implementation does not suffer from this shortcoming. Alternative approaches have also been explored, as highlighted in a recent study by Kilic et al. [74] which developed an alternative approach based on a Bayesian non-parametric method for network inference combined with different Monte Carlo samplers, particularly applied to gene expression models with an unknown number of hidden promoter-states from snapshot RNA data.

When quantifying the performance of an inference framework, it is standard procedure to perform benchmarking based on synthetic data, i.e., to perform self-consistent tests, as this approach allows us to compare to the ground truth, e.g., [52,57,62,75]. Thus, to test SLInG while avoiding *systematic* errors that arise from model misspecification [76], we provide the sampler with the same modelling libraries that are used to generate the synthetic data in the CLE-synthetic case study. However, when interpreting these benchmarking results, one must bear in mind that a self-consistent test is an idealised situation and that all models are simple abstractions of real phenomena. Whether a good performance in self-consistent tests of simulation-based approaches translates into a good performance when dealing with real-world data, therefore, depends on how well the mathematical model captures the phenomenon in question. Moreover, the choice of distance measure can contribute to reducing the impact of model misspecification [77]. To address this concern directly, the Benchmarking on real mouse embryonic stem cell scRNA-seq (BEELINE) section reports a benchmark on real BEELINE mouse embryonic stem cell scRNA-seq data where SLInG, GENIE3 and GRNBoost2 all face the same model-misspecification challenge; the margin by which SLInG outperforms GENIE3 and GRNBoost2 there is comparable to the margin reported on the self-consistent benchmark, indicating that the original conclusion is not an artefact of the self-consistent evaluation.

Several recent network-inference tools target overlapping but distinct parts of the single-cell regulatory landscape. PHOENIX [78] frames regulatory dynamics as a biologically informed neural ODE and is designed to scale to genome-wide transcription, trading off mechanistic resolution for coverage. SCENIC [79] combines co-expression-based GRN inference with cis-regulatory motif enrichment to identify transcription-factor regulons across heterogeneous cell populations. SCORPION [80] integrates transcriptomic similarity with protein-protein interaction and transcription-factor binding priors to reconstruct tissue-wide networks. These approaches exploit different sources of evidence and operate at a genome-wide scale, whereas SLInG works from a mechanistic stochastic model at the resolution of a small regulatory subnetwork. SLInG is thus complementary to this broader ecosystem: it is well suited for re-scoring candidate subnetworks flagged by a genome-scale method, for quantifying posterior uncertainty on individual interactions, and for combining data types within a single generative model.

In sparse regression, setting the level of sparsity is a difficult task. Methods such as cross-validation can be used, but they are very time-consuming [81]. To address this issue, SLInG entails hyper-parameters that adjust the sparsity-inducing prior for each parameter (similar to adaptive LASSO and SLOPE [21,24,43]) and a tuning parameter ($\delta_{ABC}$) that affects the quality of the fit to data and hence the overall level of sparsity. The decision about the level of sparsity is ultimately closely related to how much we believe in the data, as complex models will have a tendency to overfit the data. Similarly, the level of sparsity is related to how much we believe in our model due to the issue of model misspecification in real-world applications discussed above. In practice, we obtain reasonable results as long as we choose $\delta_{ABC}$ to be relatively small in our case studies. Also, the value of each hyper-parameter gives a clear indication of which parameters should be set identically to zero. Overall, this makes SLInG efficient and powerful in inferring models with the right level of complexity.

With regards to applications of SLInG to real-world data, we have recently successfully applied SLInG to reverse engineer a small cell fate genetic network based on single-molecule FISH transcript data, using seam-cells of nematodes (Caenorhabditis elegans). The combined experimental and theoretical analysis of this work is presented in a separate paper [82]. Here, we have also included the application to real scRNA-seq mESC data from the BEELINE benchmark. To extend applications of SLInG to real single-cell RNA-sequencing data for inference of larger genetic networks, we need to overcome a few challenges. Firstly, single-cell RNA-sequencing data is sparse and suffers from so-called zero-inflation [83]. As we have recently shown, one could use imputation methods to overcome this issue with varied levels of success [55]. Alternatively, one can build a model of zero-inflation, such as binomial down-sampling, into our mechanistic model and directly use the modified model that incorporates the model of technical noise for inference [84–87]. The second major problem for the inference of large genetic networks based on single-cell RNA-sequencing data is the limits of scalability of our method as discussed above. However, we can use the current framework to validate subnetworks inferred from large methods. Indeed, using an amortized inference framework [6], the inference of small subnetworks can be applied in a scalable manner to genome-scale problems as we have done in our recent work [85]. This suggests a natural division of labour in a real-data workflow: a scalable correlation-based or machine-learning method (such as GENIE3, GRNBoost2, PHOENIX or SCENIC) is first applied at the genome scale to nominate candidate links, and SLInG is then applied to small subnetworks of biological interest to re-score the nominated interactions using a mechanistic generative model and to attach posterior uncertainty to individual edges. The scalable method answers which edges are worth considering while SLInG answers which of those edges are supported by the data under a mechanistic model, and the two steps together retain the scale advantages of the first and the mechanistic interpretability of the second. Since snap-shot gene expression data is not sufficient for full identifiability of the topology and parameters of the genetic networks, we cannot expect a complete recovery from our method. However, as SLInG is using mechanistic models, we can extend our analysis easily to systematically take the experimental design into account and combine multiple types of data such as single-cell, bulk, temporal and perturbation gene expression data to improve inference of the underlying networks. Correlation-based inference methods for genetic networks, such as GENIE3 and GRNBoost2, are computationally efficient and scale well with the size of the networks, but they are not very flexible for the integration of different kinds of data. Indeed, a very promising area of future work is harnessing the information contained in multi-modal single-cell data, such as gene expression and chromatin accessibility, using inference based on mechanistic models [57,85,86,88–90]. For practitioners applying SLInG to real data, three practical considerations matter. First, initialising each chain at a partial correlation-based warm start (for example, the output of ppcor thresholded at a modest magnitude) shortens the burn-in phase substantially compared to starting from zero, particularly when the data matrix is only moderately informative about the covariance structure. Second, because the posterior geometry on sparse regulatory problems can be multimodal, we recommend running several independent chains from different initialisations and reporting results only for edges that are recovered consistently across chains; discordance between chains is itself diagnostic of a weakly identifiable parameter. Third, because SLInG is generative, posterior predictive checks in which the fitted model is forward-simulated and its summary statistics are compared to the observed data provide a natural goodness-of-fit diagnostic; a fit that fails the posterior predictive check should be treated with caution even if the chain has converged in the usual diagnostic sense.

In this paper, we demonstrate that SLInG is a flexible framework that is well-equipped to perform sparse inference based on a wide variety of simulations, including non-linear and stochastic models. Similar to some recently developed tools for parameter inference (e.g., PESTO [91,92]), SLInG is broadly applicable to black-box models, and can, therefore, be integrated with existing tools for generating models in systems biology (e.g., rule-based modelling methods such as BioNetGen [93]) and beyond to do large-scale model selection and model discovery. Not only does the presented hierarchical Bayesian sampling scheme thus provide an alternative to other algorithms, but it is also a versatile tool that can be used to address multiple research questions in systems biology and beyond.

## Supporting information

S1 File Supporting information. Section A gives a summary of the SLInG algorithm in pseudo-code. Section B contains the supporting methods: the distance measures, tuning parameters and burn-in (B.1); the settings for the linear model (B.2); the ODEs describing the signalling networks (B.3) and the corresponding settings (B.4); the stochastic differential equations describing the gene regulatory networks (B.5) and the corresponding settings (B.6); details of the GENIE3 and GRNBoost2 comparison (B.7); and the software and package versions used (B.8). Section C contains supporting Figs S1–S5.
(PDF)
