# Supplementary material for: Bayesian model discovery for reverse-engineering biochemical networks from data
Source: PLoS One. 2026 Aug 20;21(8):e0355545. doi: 10.1371/journal.pone.0355545 (PMC13492775; doi:10.1371/journal.pone.0355545)
Supplement: Supplementary file 1 [file pone.0355545.s001.pdf]

Bayesian model discovery for reverse-engineering biochemical networks from data (supporting information)

Andreas Christ Sølvsten Jørgensen[1,2,3*], Marc Sturrock[4], Atiyo Ghosh[1], Vahid Shahrezaei[1*]

1 Department of Mathematics, Imperial College London, Exhibition Rd, South Kensington, London SW7 2BX, UK

2 I-X Centre for AI In Science, Imperial College London, White City Campus, 84 Wood Lane, London W12 0BZ, UK

3 Laboratoire des Sciences du Climat et de l'Environnement, CEA CNRS UVSQ, Orme des Merisiers, 91191 Gif-sur-Yvette, France

4 Department of Physiology and Medical Physics, Royal College of Surgeons in Ireland, Dublin D02 YN77, Ireland

* Correspondence to be addressed to a.joergensen@imperial.ac.uk and v.shahrezaei@imperial.ac.uk

A Algorithm overview

Algorithm 1 Sparse likelihood-free inference using Gibbs (SLInG)

Initialize $\epsilon_{k,0}$ and $\theta_{k,0}$ $\forall k$.

Generate model and store model predictions as s_{mod}.

Compute the distance, d_0, between the model prediction and observations.

$\delta_{\text{ABC}} = \max(\delta_{\min}, d_0/\delta_{\max})$.

for $i \leq N$ **do**

  Randomly shuffle the order of $\epsilon_{k,i}$ and $\theta_{k,i}$

  if i>grace period **then** ▷ Initially, we just want to probe the parameter space.

    for $k \leq K$ **do**

      Sample $\epsilon_{k,i}$ from the conditional posterior, i.e evaluate the distribution of $\epsilon_{k,i}$ that could have led to $\theta_{k,i-1}$ and draw from this distribution after considering priors on $\epsilon_{k,i}$.

    end for

  end if

  for $k \leq K$ **do**

    if i>burn-in/2. **then**

      Reset σ_{s}^2 to the variance of θ_k in previous samples. If zero, use the default value.

    end if

    Sample $\theta^*_{k,i}$ from (normal) proposal distribution, q, centered at $\theta_{k,i-1}$: $q = \mathcal{N}(\theta_{k,i-1}, \sigma_{\text{s}}^2)$

    Generate model prediction, s_{sim}, from new parameter set.

    Compute distance, $d(s_{\text{sim}}, s_{\text{obs}})$ between the model predictions and observations, s_{obs}.

    Compute α based on d, q and the sparsity prior:

$$\alpha = \frac{\psi(d(s_{\text{sim}}, s_{\text{obs}})/\delta_{\text{ABC}})\pi(\theta_{k,i}|\epsilon_{k,i})q(\theta_{k,i}|\theta_{k,i-1})}{\psi(d(s_{\text{mod}}, s_{\text{obs}})/\delta_{\text{ABC}})\pi(\theta_{k,i-1}|\epsilon_{k,i-1})q(\theta_{k,i-1}|\theta_{k,i-2})}, \qquad \psi = \mathcal{N}(0,1)$$

    Draw $p^* \sim U(0,1)$

    if $p^* < \alpha$ **then**

      Store $\theta_{k,i} \leftarrow \theta^*_{k,i}$

      Store $s_{\text{mod}} \leftarrow s_{\text{sim}}$

    else

      Store $\theta_{k,i} \leftarrow \theta_{k,i-1}$

    end if

  end for

end for

The algorithm might be called several times during the burn-in phase to iteratively adjust δ_{ABC} to the value set by the user ($\delta_{\min}$). The grace period is only included during the first of these iterations. During the grace period, $\epsilon_{k,i}$ is set to 1 for all k. Once i exceeds the grace period, the hyper-parameter update runs on every subsequent iteration of the outer loop rather than only on the first, so that each $\epsilon_{k,i}$ tracks

the current state of $\theta_{k,i-1}$ throughout the main sampling phase.

The conditional posterior sampled for each hyper-parameter in Algorithm 1 is obtained by treating $\theta_{k,i-1}$ as fixed and applying Bayes' rule to the Laplace prior of equation (**??**) together with a prior on $\epsilon_{k,i}$ itself, giving

$$p(\epsilon_{k,i} \mid \theta_{k,i-1}) \propto \pi(\theta_{k,i-1} \mid \epsilon_{k,i})\, \pi(\epsilon_{k,i}) = \frac{\epsilon_{k,i}}{2} \exp(-\epsilon_{k,i}|\theta_{k,i-1}|)\, \pi(\epsilon_{k,i}). \tag{1}$$

We evaluate this density on a dense grid of $\log_{10}(\epsilon_{k,i})$ values and draw $\epsilon_{k,i}$ from the resulting discrete distribution. A uniform prior on $\log_{10}(\epsilon_{k,i})$ is used in all case studies, so that $\pi(\epsilon_{k,i})$ sets the grid range and the update is driven by the current value of $\theta_{k,i-1}$.

B Supplementary methods

B.1 Distance measures, tuning parameters and burn-in

The likelihood is often intractable when dealing with models that describe real-world dynamics. ABC methods, such as SLInG, circumvent this issue by introducing distance measures (d) that quantify the proximity between the data and the predictions of mathematical models [cf. 13]. For this purpose, ABC methods rely on suitable summary statistics $\mathbf{s} = (s_1, s_2, \ldots, s_N)$ that capture information in simulated and observed data sets. What constitutes an appropriate distance measure is thus determined by the scientific question that one aims to address and by the data that one aims to fit. In the paper, we encounter three very distinct case studies, and we will define the appropriate distance metric in the corresponding subsections of the result section. To clarify the terminology, we note that the introduction of a distance measure means that ABC methods are said to be likelihood-free. However, when introducing the distance measure in the kernel of our Monte Carlo sampling scheme, we effectively introduce a surrogate likelihood [e.g. 12].

The sparsity of the fit is determined by δ_{ABC}. If δ_{ABC} is too high, changes in d have little or no impact on the acceptance rate, i.e. the priors dominate the posterior, making the solution artificially sparse. On the other hand, if δ_{ABC} is too low, the Markov chain acceptance rate plummets, hindering convergence (see also Section 2.3 in the paper). We thus explore, quantify and discuss the impact of our choice of δ_{ABC} for each case study.

At the beginning of each new step in the Markov chain, we calibrate ϵ_k separately for each θ_k. For this purpose, we consider a large number of uniformly distributed values of $\log_{10}(\epsilon_k)$. For each of these values, we compute the probability of obtaining the current value of θ_k from the sparsity prior. We then randomly sample a new value of ϵ_k from the resulting distribution. In short, we sample ϵ_k from the conditional posterior based on the previous step (cf. supporting information section A). In this manner, ϵ_k is iteratively adjusted: If the exploration of the parameter space yields a low value of θ_k, a large value of ϵ_k will be imposed when using the Laplace prior, which further drives θ_k to zero unless the resulting changes in the distance measure provide evidence to the contrary. On the other hand, if the data points to a large value of θ_k, ϵ_k will be small when using the Laplace prior, relaxing the imposed sparsity for this particular parameter.

In all case studies, we start our exploration of the parameter space from generic or randomised initial conditions. As a result, we need to discard a burn-in phase. During this burn-in phase, δ_{ABC} must be set to a sufficiently high value to allow sufficient exploration of the parameter space by smoothing out the impact that parameter changes have on the distance measure. Otherwise, the Gibbs sampler might get stuck in an excessively sparse solution. In other words, during the burn-in phase, δ_{ABC} might need to decrease to the desired value in a step-wise manner. Moreover, as a part of the burn-in, we introduce an initial grace period, during which the hyper-parameters are kept at low values, to allow a proper exploration of the parameter space.

It is useful to distinguish clearly between these two concepts. The grace period is a short exploratory phase at the very start of the first burn-in call in which the hyper-parameters ϵ_k are held fixed at a low value (set to 1 in all case studies), so that the sparsity prior contributes only weakly to the acceptance

rate and the chain can move freely to probe the broad shape of the parameter space. The burn-in is the standard MCMC convergence phase that follows, during which the hyper-parameter update of equation (1) is switched on and δ_{ABC} may be tightened iteratively towards $\delta_{\min}$. The two phases play complementary rather than duplicative roles: the grace period prevents the chain from collapsing onto a spuriously sparse corner before it has had a chance to locate the bulk of the posterior, while the burn-in gives the sparsity-inducing hyper-parameters time to adapt to the parameter values that the chain eventually visits. Samples drawn before the end of the burn-in are discarded for subsequent analysis.

Besides δ_{ABC}, the width (σ_{s}) of the proposal distribution (q) determines the acceptance rate and sets the conditions for the exploration of the parameter space (cf. supporting information section A). If the starting point of the Markov chain is far from the true parameter values, the proposal distribution should be broad. On the other hand, the parameter space around the best fit should be properly explored, requiring a narrow proposal distribution. Thus, σ_{s} must be iteratively adjusted during the run based on the previous samples. σ_{s} plays an analogous role to an adaptive learning rate in that it controls the steps size of the parameter exploration.

Finally, to interpret the results obtained from the sampler, we need to make one more choice: If a parameter is superfluous, we obtain a narrow posterior distribution for the parameter around zero. Like for other sparse *Bayesian* inference methods, redundant parameters are not directly set to zero but take values close to zero. We thus need to choose, e.g., a threshold beyond which we interpret the parameter as being discarded by SLInG. Since this choice is problem-dependent, it is addressed separately for each individual application below (cf. Sections B.2, B.4 and B.6).

B.2 Linear models, settings

We use the diabetes dataset from Efron et al. provided through Scikit-learn [11].

We fit the data set from Efron et al. using four different values of δ_{ABC}. In all four cases, we impose truncated Laplace priors, restricting parameters to values between -1,000 and 1,000.

We construct 100 synthetic data sets. To create a synthetic data set, we randomly select between 1 and 6 of the 10 parameters and let these parameters take random values between 50 and 900. We then compute the resulting model response using our linear model. Finally, to ensure a realistic behaviour of the synthetic data, we add 5 percent Gaussian noise to each data point, i.e. each data point x_j in the original synthetic data set is substituted by $x_j^{\mathrm{obs}} \sim \sqsupseteq(\mu = x_j, \sigma^2 = 0.05^2 x_j^2)$. The 5 percent level was chosen as a compromise between two regimes: smaller values recover an essentially noise-free regression problem that does not exercise the sparsity prior, while substantially larger values would dominate the variation of the response variable and obscure the contribution of any individual predictor. A 5 percent multiplicative noise is also representative of the residual variation observed in the original Efron et al. [1] data set once the strongest predictors are regressed out.

To map the impact of δ_{ABC}, we repeat the inference for each of the 100 data sets using 66 different values of δ_{ABC} between 0.00125 and 0.05. We thus perform 6,600 separate inference tasks using the same truncated Laplace priors as in the case of the real-world data. For this analysis, we consider any parameter that takes a value lower than 10 as discarded since the relevant parameter range stretches from 50 to 900.

B.3 Adaptation, ODEs

The considered adaptive regulatory networks [see also 8] are described by a system of three ordinary differential equations (ODEs):

$$\frac{\mathrm{d}A}{\mathrm{d}t} = f(k_0, K_0, A, A) + f(k_1, K_1, A, B) + f(k_2, K_2, A, C) + f(k_3, K_3, A, E_A) + f(k_4, K_4, A, I), \quad (2)$$

$$\frac{\mathrm{d}B}{\mathrm{d}t} = f(k_5, K_5, B, A) + f(k_6, K_6, B, B) + f(k_7, K_7, B, C) + f(k_8, K_8, B, E_B), \quad (3)$$

$$\frac{\mathrm{d}C}{\mathrm{d}t} = f(k_9, K_9, C, A) + f(k_{10}, K_{10}, C, B) + f(k_{11}, K_{11}, C, C) + f(k_{12}, K_{12}, C, E_C), \quad (4)$$

where A, B, and C denote the three genes/enzymes in the regulatory network, while E_A, E_B, and E_C are other activating/deactivating enzymes that might affect A, B, or C, respectively. I denotes the input signal, and k_0-k_{12} and K_0-K_{12} are the 26 free parameters that we aim to fit. Finally, f denotes the Hill function

$$f(k, K, G_1, G_2) = G_1 k \left(\frac{H(k) - H(k)B + H(-k)B}{H(k) - H(k)B + H(-k)B + K} \right) \quad (5)$$

where $H(k)$ denotes the Heaviside function, i.e. the sign of k determines whether the link leads to repression or activation.

B.4 Adaptation, settings

For all parameters in equations (2-4) in supporting information section B.3, we use uniform priors. We allow k_0-k_{12} (with the exception of k_4) to take values between -10 and 10, i.e. all links might either be repressive or activating. We allow $K_0 - K_{12}$ as well as k_4 to vary between 0.1 and 10. The reason we single out k_4 is that we only consider networks in which the input activates the first node (A).

We note that there must be both positive and negative terms on the right-hand side of Equations 2-4 since the concentration of A, B, and C will otherwise diverge. Thus, we discard all models for which the sum over the negative link strengths or the sum over positive link strengths falls below a protein-specific threshold, i.e. the Markov chain does not pass through such points. For A and C, we set the threshold to 10^{-3}, while we set the threshold to 10^{-4} when dealing with B. These thresholds are somewhat arbitrary. Changing them would further limit or expand the parameter space that we explore. We also note that B is treated differently since it's a buffer node/proportioner: According to Ma et al., the presence of such nodes is essential for achieving adaptation, making us more reluctant to discard links to B.

We set up one hundred runs starting each run from a fully connected network with uniform link strengths of 0.1. Each of the one hundred runs are subdivided into groups of twenty, with each group using a different value of δ_{ABC}: 0.5, 0.2, 0.1, 0.05, and 0.029. The lastwvalue (1/35) in this list is determined by the distance measure of the initial conditions and is, hence, the lowest possible value for our set-up (we set $\delta_{\max} = 35$, cf. supporting information section A). For each run, we create a single Markov chain with 10,000 samples.

After each run, we extract the last 4,000 models in the Markov chain and set all link strengths that fall below a certain threshold to zero. We vary this threshold, increasing it from 10^{-3} to 10^{-2} and, finally,

to 10^{-1}. We then recompute P and S for each of the models, as both P and S will change when setting some parameters to zero. We then identify adaptive networks, i.e. networks with $S > 1$ and $P > 10$.

Among the 31 successful topologies, δ_{ABC} was set to 0.029, 0.05 and 0.1 in 7, 18 and 6 cases, respectively, while no adaptive systems were found when choosing higher values of δ_{ABC}.

B.5 Stochastic regulatory genetic networks, SDEs

The considered stochastic regulatory genetic networks are represented by a system of eight stochastic differential equations (SDEs). These were obtained using the framework of chemical Langevin equations [3, 7]:

$$\begin{aligned} d[m_1] =& (p_2a_1k_{11}k_6k_5 + p_3a_2k_{11}k_6k_5 + p_4a_3k_{11}k_6k_5 + k_{11} - (k_2+\mu)[m_1])\,dt \\ &+ \sqrt{p_2a_1k_{11}k_6k_5}dW_1 + \sqrt{p_3a_2k_{11}k_6k_5}dW_2 + \sqrt{p_4a_3k_{11}k_6k_5}dW_3 \\ &+ \sqrt{k_{11}}dW_{13} - \sqrt{(k_2+\mu)[m_1]}dW_{14}, \end{aligned} \tag{6}$$

$$\begin{aligned} d[m_2] =& (p_1a_4k_{12}k_6k_5 + p_3a_5k_{12}k_6k_5 + p_4a_6k_{12}k_6k_5 + k_{12} - (k_2+\mu)[m_2])\,dt \\ &+ \sqrt{p_1a_4k_{12}k_6k_5}dW_4 + \sqrt{p_3a_5k_{12}k_6k_5}dW_5 + \sqrt{p_4a_6k_{12}k_6k_5}dW_6 \\ &+ \sqrt{k_{12}}dW_{17} - \sqrt{(k_2+\mu)[m_2]}dW_{18}, \end{aligned} \tag{7}$$

$$\begin{aligned} d[m_3] =& (p_1a_7k_{13}k_6k_5 + p_2a_8k_{13}k_6k_5 + p_4a_9k_{13}k_6k_5 + k_{13} - (k_2+\mu)[m_3])\,dt \\ &+ \sqrt{p_1a_7k_{13}k_6k_5}dW_7 + \sqrt{p_2a_8k_{13}k_6k_5}dW_8 + \sqrt{p_4a_9k_{13}k_6k_5}dW_9 \\ &+ \sqrt{k_{13}}dW_{21} - \sqrt{(k_2+\mu)[m_3]}dW_{22}, \end{aligned} \tag{8}$$

$$\begin{aligned} d[m_4] =& (p_1a_{10}k_{14}k_6k_5 + p_2a_{11}k_{14}k_6k_5 + p_3a_{12}k_{14}k_6k_5 + k_{14} - (k_2+\mu)[m_4])\,dt \\ &+ \sqrt{p_1a_{10}k_{14}k_6k_5}dW_{10} + \sqrt{p_2a_{11}k_{14}k_6k_5}dW_{11} + \sqrt{p_3a_{12}k_{14}k_6k_5}dW_{12} \\ &+ \sqrt{k_{14}}dW_{25} - \sqrt{(k_2+\mu)[m_4]}dW_{26}, \end{aligned} \tag{9}$$

$$d[p_1] = (k_3[m_1] - (k_4+\mu)[p_1])\,dt + \sqrt{k_3[m_1]}dW_{15} - \sqrt{(k_4+\mu)[p_1]}dW_{16}, \tag{10}$$

$$d[p_2] = (k_3[m_2] - (k_4+\mu)[p_2])\,dt + \sqrt{k_3[m_2]}dW_{19} - \sqrt{(k_4+\mu)[p_2]}dW_{20}, \tag{11}$$

$$d[p_3] = (k_3[m_3] - (k_4+\mu)[p_3])\,dt + \sqrt{k_3[m_3]}dW_{23} - \sqrt{(k_4+\mu)[p_3]}dW_{24}, \tag{12}$$

$$d[p_4] = (k_3[m_4] - (k_4+\mu)[p_4])\,dt + \sqrt{k_3[m_4]}dW_{27} - \sqrt{(k_4+\mu)[p_4]}dW_{28}, \tag{13}$$

where $[m_1]$ to $[m_4]$ represent mRNA concentrations for each gene, while $[p_1]$ to $[p_4]$ represent protein concentrations for each gene. The terms dW_1 to dW_{28} are independent Wiener processes, with each process representing the stochastic noise from one of the 28 underlying chemical reactions. Finally, activation or repression interactions are represented by

$$h(p,a,K,n) = \begin{cases} \frac{ap^n}{p^n+K^n} & \text{if } a > 0 \\ \frac{aK}{p+K} & \text{if } a < 0 \end{cases}$$

where k_{11}, k_{12}, k_{13}, and k_{14} represent the basal transcription rates for each of the four genes, respectively. Moreover, k_2 represents the rate of mRNA decay, k_3 represents the translation rate, and k_4 is the rate of protein decay. Cell dilution is modelled to occur as a result of exponential cell growth and is characterised by the rate μ, while k_5 is a Hill coefficient, and k_6 is a dissociation constant. Sparse genetic networks can be created by setting the link strength parameters, a_1 to a_{12}, to zero.

We note that the impact of the strengths of the different regulatory links on mean gene expression can be mimicked by changing the base rates of gene expression for the four genes (i.e. $k_{11} - k_{14}$). To this end, the base rates and the link strengths are degenerate. In real-world applications, $k_{11} - k_{14}$ are unknown and must hence be inferred alongside the strength of the different links between the genes. We thus require SLInG to infer $k_{11} - k_{14}$ as well as 12 parameters, one associated with the possible links between the genes, i.e. we have 16 free parameters in total.

Because we do not impose any sparsity priors on $k_{11} - k_{14}$ (cf. supporting information section B.6), the sampler increases $k_{11} - k_{14}$ above the ground truth to reduce the link strengths and potentially discard links. This behaviour is due to the mentioned degeneracy between the impact of $k_{11} - k_{14}$ and the link strengths on the mean gene expression. SLInG thus points to the existence of links based on the contribution of the covariance matrix to the distance measure rather than based on the mean gene expression.

B.6 Stochastic regulatory genetic networks, settings

For the 12 link strengths, we impose truncated Laplace priors, constraining them to take values between -20 and 20: Negative link strengths imply that the corresponding link is repressive, while positive links lead to activation. For $k_{11} - k_{14}$, on the other hand, we do not impose sparsity priors but impose uniform priors on their logarithm from -2 to 2. The choice of ± 20 for the link-strength bounds is dictated by the dynamic range over which the Hill-type interaction term is effectively saturated given the parameter scales used here. Absolute link strengths above the upper bound push the regulated mRNA production close to its asymptotic ceiling (or floor, for repressive links) so that further increases produce negligible changes in steady-state expression, while the ground-truth values used to construct the synthetic data lie within $[3, 10]$ and therefore sit safely inside $[-20, 20]$. Widening the range has no measurable effect on the inference—narrowing it starts to truncate the Laplace prior artificially for the strongest interactions.

We created 100 synthetic data sets based on random 4-node gene-regulatory networks. For each data set, we randomly selected the total number of existing (non-zero) links, the number of repressive and adaptive links amongst the non-zero links, and the strength of each of the non-zero links. Thus, each synthetic data set stems from a network with between 3 and 5 non-zero links that were each randomly set to be repressive or activating, with both choices equally likely. The absolute strength of the links all take values between 3.0 and 10.0, i.e. safely within the prior of the sampler. Because the detection of links relies on the contribution of the covariance matrix to the distance measure, we further required that all synthetic data sets show significant correlations between the gene expressions. We thus only adopted a randomly chosen parameter set into our library of synthetic data sets if at least six out of the twelve off-diagonal elements in the *correlation* matrix of the resulting synthetic data were larger than 0.3. Otherwise, we tried another parameter set. In other words, the 100 synthetic data sets are selected using trial and error to fulfil our condition on the correlation matrix. For all runs, $k_{11} - k_{14}$ are set to 0.40, 0.37, 0.34 and 0.30, respectively.

In the paper, we increase the value of δ_{ABC} to make the sampler less sensitive to the stochasticity of the simulations. More specifically, we found that a value of 0.15 allows our sampler to efficiently explore the parameter space while still capturing the essential information in the data for most parameter sets.

In some cases, we found that the chain still seemed to get stuck. If we were dealing with real-world data, we would try different values of δ_{ABC} to fit the data. However, rather than choosing a larger value for δ_{ABC} for these cases, we simply discarded the synthetic data set, created a new one, and repeated the analysis. This choice was made to ensure a uniform value of δ_{ABC} across the samples.

To infer the parameter values that underlie the synthetic data, we initialised each run, setting all parameters to zero, including the logarithm of $k_{11} - k_{14}$. For each of the 100 runs, we computed 5,500 samples. Each sample involves 16 simulations, as we are exploring a 16-dimensional parameter space. The initial 1,500 samples functioned as a burn-in phase, which allowed us to adjust δ_{ABC} iteratively. For the analysis of each run, we focus on the best-fitting model amongst the 4,000 samples that were left after excluding this burn-in phase. As a first quality check, we determine whether the best-fitting parameter set produces gene expressions that lie sufficiently close to the data. This comparison is performed in three steps: First, we bootstrap the observations, i.e. the synthetic data, and compute the distance of each bootstrapped data set to the observations. Secondly, we compute the gene expression of the best-fitting parameter set after discarding any links with strengths below 10^{-1}. This cut-off was set based on the following considerations: we only want to keep the most prominent links. However, while no data set is constructed with a link strength lower than 3.0, the inference will yield lower values due to the aforementioned degeneracy between the link strengths and $k_{11} - k_{14}$.

Thirdly, we bootstrap the results for the best-fitting model, computing the distance between each bootstrapped set of predictions and the observations. If the resulting distribution of distances overlaps with even one sample from the distribution of the distance between the bootstrapped data and the observations from the first step of our comparison discussed above, we accept the fit. Otherwise, we discard the fit. Were we forced to discard a fit when applying the algorithm to real-world data, we could try to improve the fit by, e.g., adjusting initial conditions. However, as mentioned in the paper, we note that the algorithm passed our quality check out of the box in 56 percent of the cases. This success rate is not significantly affected by the cut-off in link strength for determining the number of non-zero links.

B.7 Other algorithms for reverse engineering signalling networks

GENIE3, an acronym for GEne Network Inference with Ensemble of trees, was the best performer at the DREAM4/DREAM5 network challenges [9], and though developed more than a decade ago, it still remains a favoured method for this kind of inference [4, 6]. The algorithm uses an ensemble of decision trees to model the expression of a gene as a function of the expression of other genes. Its primary assumption is that the expression of a gene is the combined effect of the expression of other genes, which is captured by the tree structure. This leads to a directed network where the genes are connected based on their inferred influence on each other.

GRNBoost2 is a more recently developed algorithm for gene regulatory network inference and is based on the GENIE3 architecture [10]. Despite sharing the same aim to uncover complex gene interactions, the two methods employ different machine-learning strategies. GRNBoost2 employs gradient boosting machines, an ensemble learning method that refines new models iteratively to provide a more precise approximation of the response variable [2]. In the context of GRNBoost2, the response variable corresponds to the expression level of a target gene. With each iteration, GRNBoost2 works to minimise the residuals

between the predicted and actual expression levels of a target gene, successively refining its model. It constructs a network by identifying the relationships between a transcription factor and a target gene that maximise shared mutual information, taking into account the effects of all other transcription factors. Although GRNBoost2 operates under a different learning method, its ultimate goal aligns with that of GENIE3: It aims to generate an accurate, directed network that represents gene regulatory relationships.

The practical implementation of GENIE3 and GRNBoost2 in large-scale network inference tasks can be computationally demanding. To overcome this issue, we made use of the Arboreto library, a highly efficient Python framework that facilitates the application of both these algorithms [10]. Arboreto has been optimised for scalability and performance. By leveraging Dask, a flexible parallel computing library for analytic computing [14], Arboreto is able to distribute and parallelise the execution of the GRN inference tasks over multiple cores or nodes. It also employs smart scheduling to prioritise the most crucial tasks, thereby maximising computational efficiency.

The application of GENIE3 and GRNBoost2 results in an ordered list of interactions ranked in terms of their inferred 'importance' or regulatory significance within the gene network. However, as these are stochastic methods, they introduce an element of randomness in their process, potentially leading to slight variations in outcomes across different runs. To mitigate this stochastic nature and provide robustness to our results, we conducted 100 repeats of the network inference process and aggregated the results by taking an average across all runs. We then used an Elbow-method to estimate the number of interactions from the full list. While GENIE3 gives us a directed network, we still would not know the sign of the regulation (activation or repression). Therefore, we used the 'ppcor' package in R to compute the sign of the interactions [5] and produce estimates of signed, directed networks that we can compare with SLInG.

B.8 Software and package versions

Table 1 lists the principal scientific software packages used to produce the results reported in this paper. The full reproducibility pipeline, including the scripts that generate the synthetic data sets and the figures, is available in the public GitHub repository at `https://github.com/ASoelvsten/SLInG`.

Package	Version	Language / ecosystem	Role in this paper
NumPy	1.26.4	Python	Array operations and random-number generation
SciPy	1.17.0	Python	ODE integration, linear algebra, statistics
Matplotlib	3.10.8	Python	Figures 2–5 and supplementary figures
scikit-learn	1.8.0	Python	Diabetes data loader (Section **??**)
Arboreto (GRNBoost2)	0.1.6	Python	GRNBoost2 baseline (Section **??**)
GENIE3	standalone	Python	GENIE3 baseline (Section **??**)
Dask	2026.3.0	Python	Parallel execution of Arboreto
PyJulia	0.6.2	Python / Julia bridge	Driver for the Julia simulator from Python
ppcor	1.1-4 (CRAN)	R	Partial-correlation sign inference for GENIE3/GRNBoost2 outputs
Catalyst.jl	16.1.1	Julia	Chemical Langevin simulation of the 4-gene networks
DifferentialEquations.jl	7.17.0	Julia	SDE integration backend for Catalyst.jl

Table 1: Principal scientific software used in this paper, with the versions that produced the reported results. The GitHub repository at `https://github.com/ASoelvsten/SLInG` contains the scripts that invoke each package.

References

[1] Bradley Efron, Trevor Hastie, Iain Johnstone, and Robert Tibshirani. Least angle regression. *The Annals of Statistics*, 32(2):407–451, 2004.

[2] Jerome H. Friedman. Greedy function approximation: A gradient boosting machine. *The Annals of Statistics*, 29(5):1189–1232, 2001.

[3] Daniel T. Gillespie. The chemical langevin equation. *Journal of Chemical Physics*, 113:297–306, 2000.

[4] Vân Anh Huynh-Thu, Alexandre Irrthum, Louis Wehenkel, and Pierre Geurts. Inferring regulatory networks from expression data using tree-based methods. *PloS one*, 5(9):e12776, 2010.

[5] Seongho Kim. ppcor: an r package for a fast calculation to semi-partial correlation coefficients. *Communications for statistical applications and methods*, 22(6):665, 2015.

[6] Ayoub Lasri, Vahid Shahrezaei, and Marc Sturrock. Benchmarking imputation methods for network inference using a novel method of synthetic scrna-seq data generation. *BMC bioinformatics*, 23(1):236, 2022.

[7] Torkel E. Loman, Yingbo Ma, Vasily Ilin, Shashi Gowda, Niklas Korsbo, Nikhil Yewale, Chris Rackauckas, and Samuel A. Isaacson. Catalyst: Fast and flexible modeling of reaction networks. *PLOS Computational Biology*, 19(10):e1011530, October 2023.

[8] Wenzhe Ma, Ala Trusina, Hana El-Samad, Wendell A Lim, and Chao Tang. Defining network topologies that can achieve biochemical adaptation. *Cell*, 138(4):760—773, August 2009.

[9] Daniel Marbach, James C Costello, Robert Küffner, Nicole M Vega, Robert J Prill, Diogo M Camacho, Kyle R Allison, Manolis Kellis, James J Collins, et al. Wisdom of crowds for robust gene network inference. *Nature methods*, 9(8):796–804, 2012.

[10] Thomas Moerman, Sara Aibar Santos, Carmen Bravo González-Blas, Jaak Simm, Yves Moreau, Jan Aerts, and Stein Aerts. Grnboost2 and arboreto: efficient and scalable inference of gene regulatory networks. *Bioinformatics*, 35(12):2159–2161, 2019.

[11] F. Pedregosa, G. Varoquaux, A. Gramfort, V. Michel, B. Thirion, O. Grisel, M. Blondel, P. Prettenhofer, R. Weiss, V. Dubourg, J. Vanderplas, A. Passos, D. Cournapeau, M. Brucher, M. Perrot, and E. Duchesnay. Scikit-learn: Machine learning in Python. *Journal of Machine Learning Research*, 12:2825–2830, 2011.

[12] Ben M. Rendle, Gaël Buldgen, Andrea Miglio, Daniel Reese, Arlette Noels, Guy R. Davies, Tiago L. Campante, William J. Chaplin, Mikkel N. Lund, James S. Kuszlewicz, Laura J. A. Scott, Richard Scuflaire, Warrick H. Ball, Jiri Smetana, and Benard Nsamba. AIMS - a new tool for stellar parameter determinations using asteroseismic constraints. *MNRAS*, 484(1):771–786, 2019.

[13] Mikael Sunnåker, Alberto Giovanni Busetto, Elina Numminen, Jukka Corander, Matthieu Foll, and Christophe Dessimoz. Approximate bayesian computation. *PLOS Computational Biology*, 9:e1002803, 01 2013.

[14] Dask Development Team. *Dask: Library for dynamic task scheduling*, 2016.

C Supplementary figures

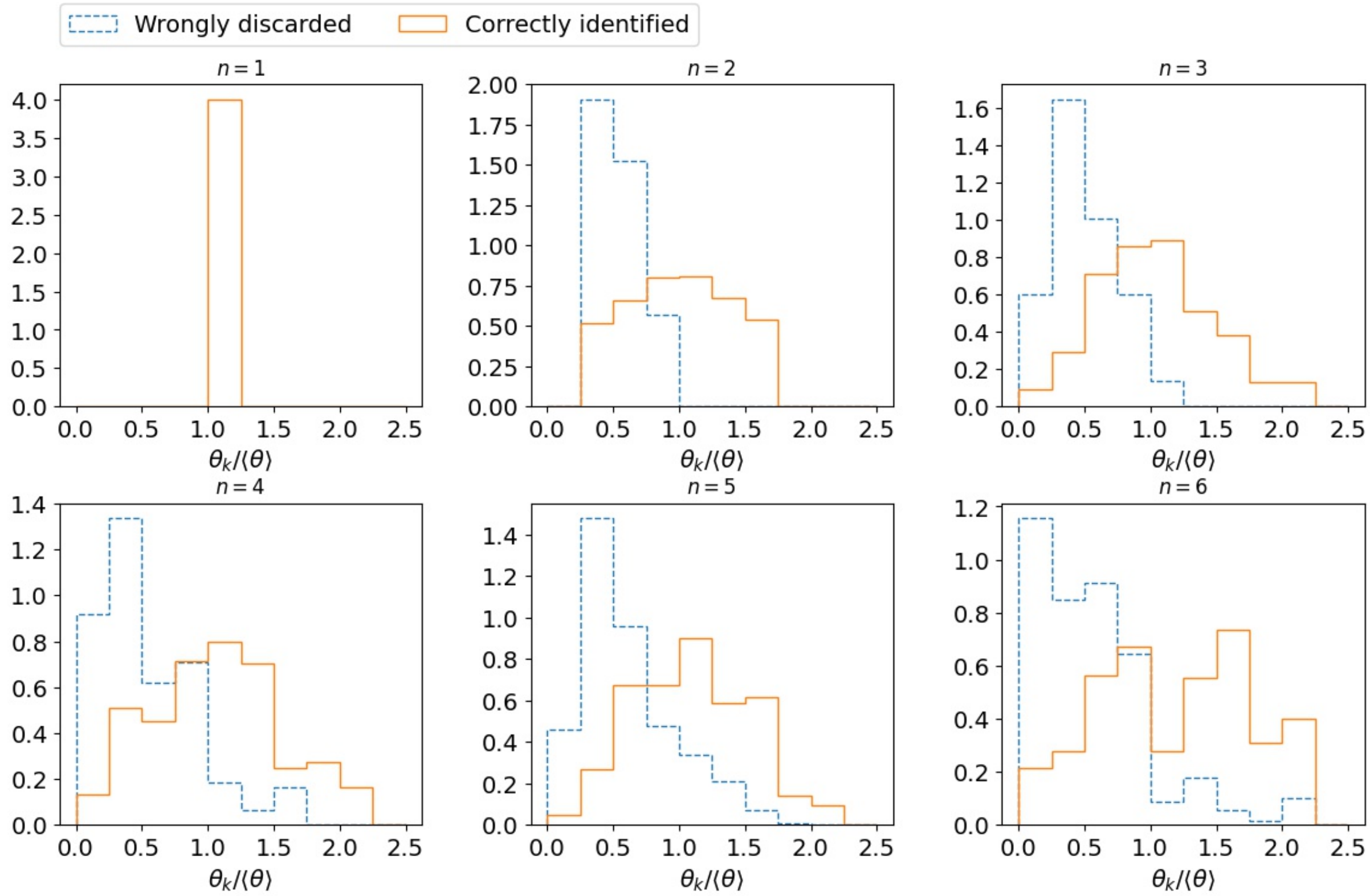

Figure S1: Parameter values across the different number of parameters (n) employed to produce the synthetic data for our linear model in Section 2.1. The plot compares the true values of the parameters that were correctly identified with the values of those that were not found by SLInG. The plot includes the same 6600 simulations presented in Fig 2 in the main text. The parameters that are not correctly identified by the algorithm tend to have the weakest impact on the response variable. They thus take values that are lower than the mean parameter values across the n true parameters ($\langle\theta\rangle$). In contrast, SLInG correctly points to the parameters with the highest explanatory power.

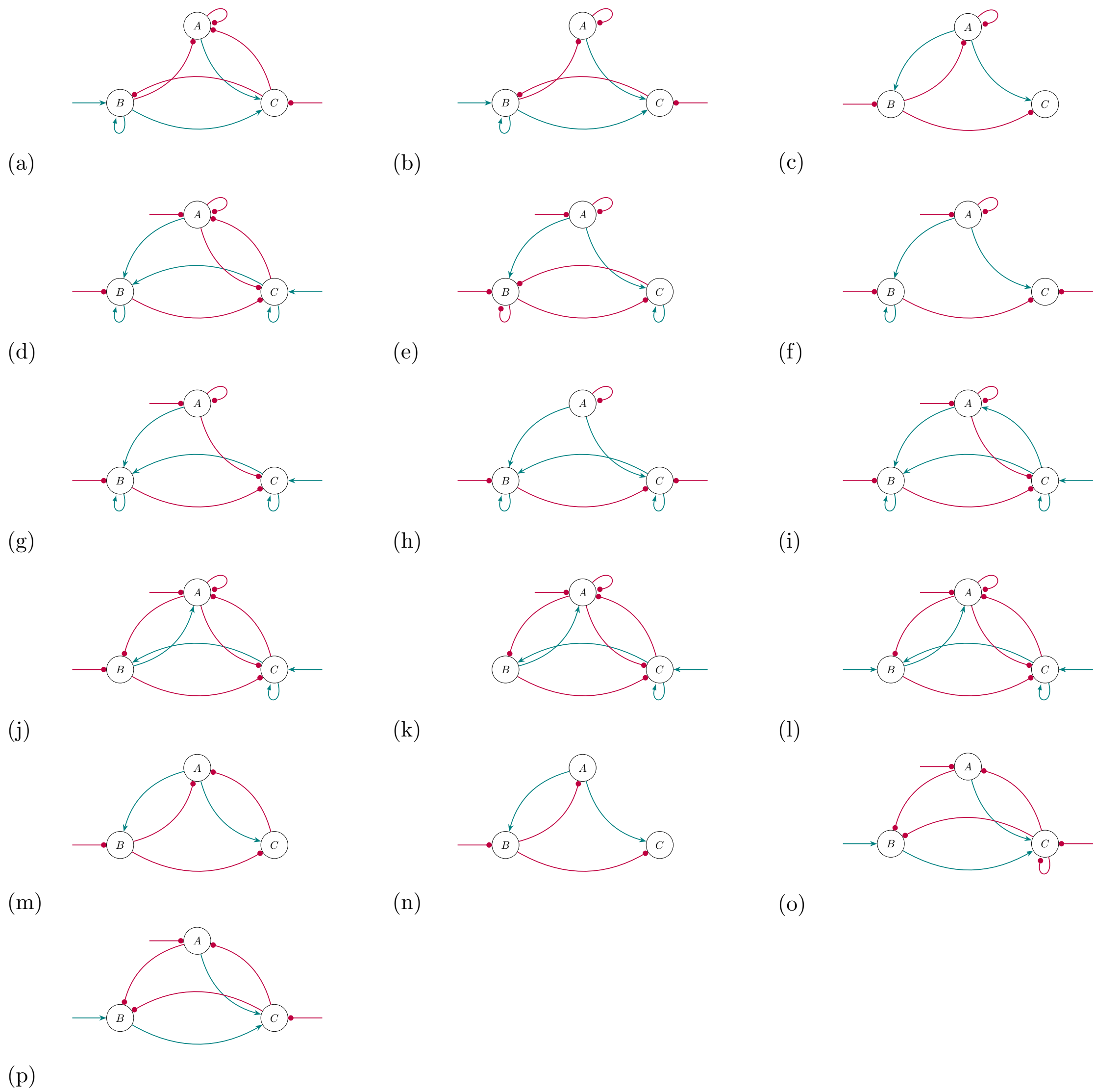

Figure S2: 16 of the 31 adaptive regulatory networks found by the sampler.

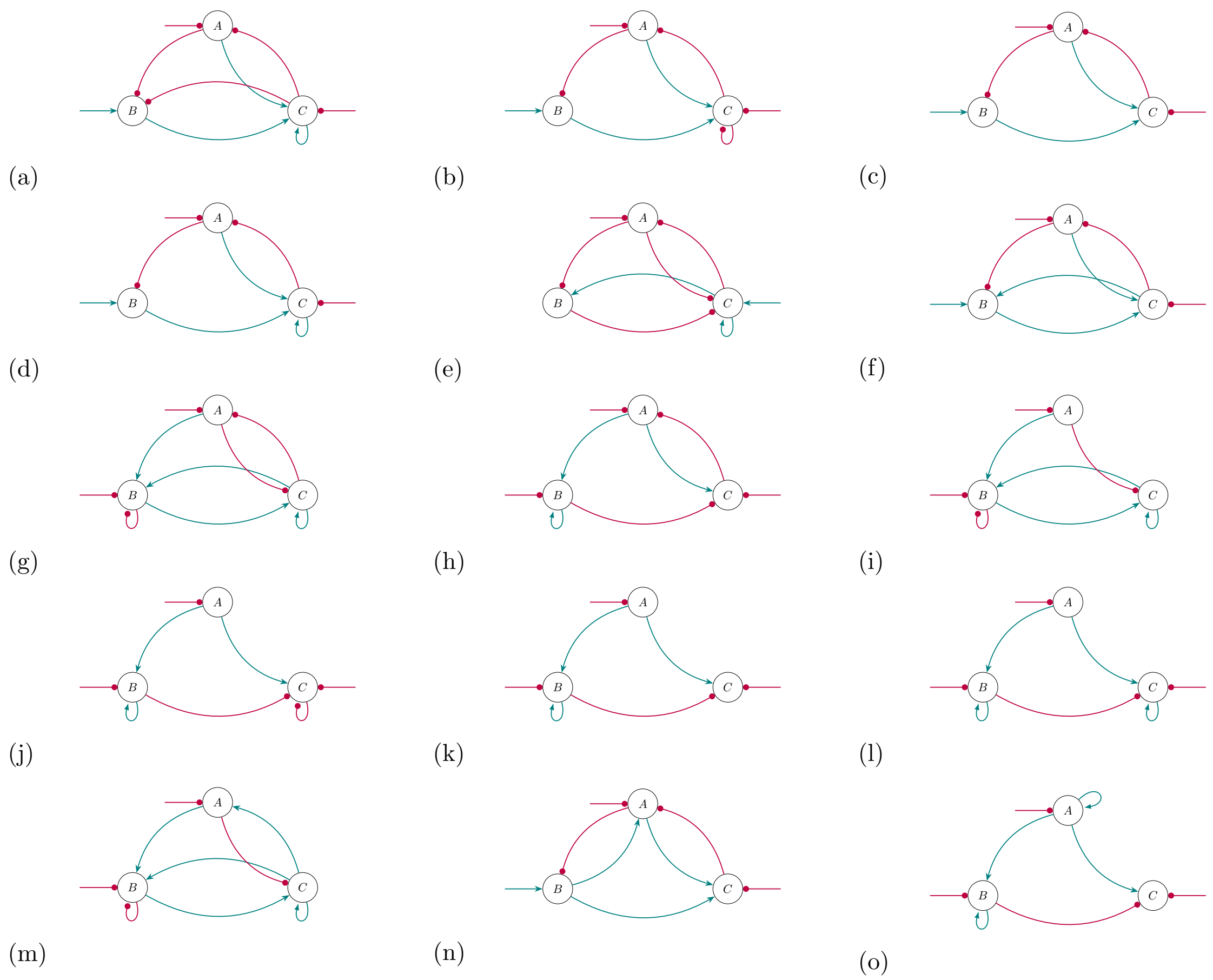

Figure S3: 15 of the 31 adaptive regulatory networks found by the sampler.

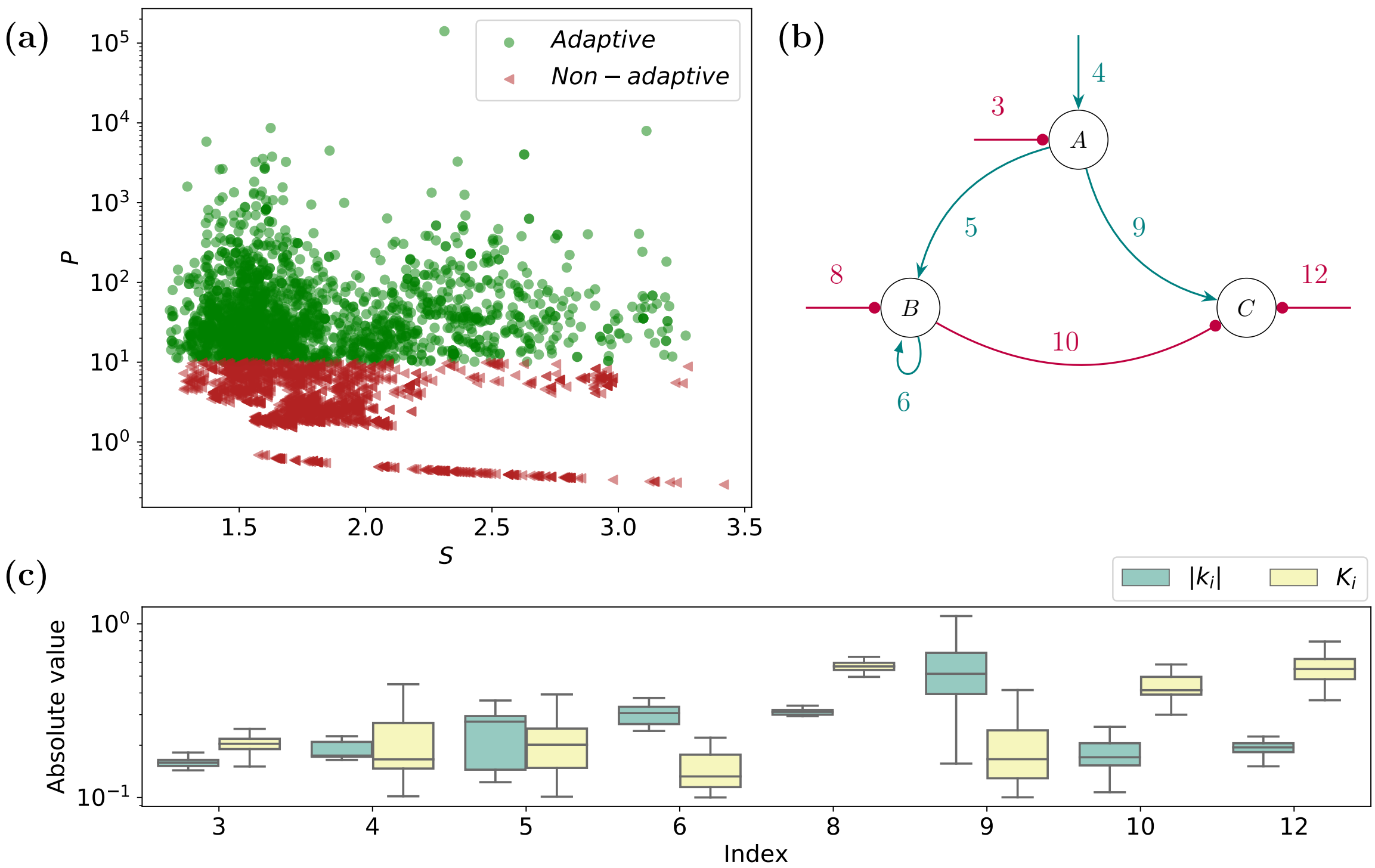

Figure S4: The impact of changing parameter values on P and S for the topology in panel (l) of Fig S3. (a) P as a function of S for all samples with this topology among the 4,000 last samples of the Markov chain. (b) The topology of the signalling network with the indices of the corresponding terms from Equations (2)-(4). (c) The parameter values *that lead to adaptation* within the samples. The index (i) corresponds to the link number in panel b. As can be seen from the figure, not all parameter combinations lead to adaptation, although adaptation is preserved for a range of parameter values.

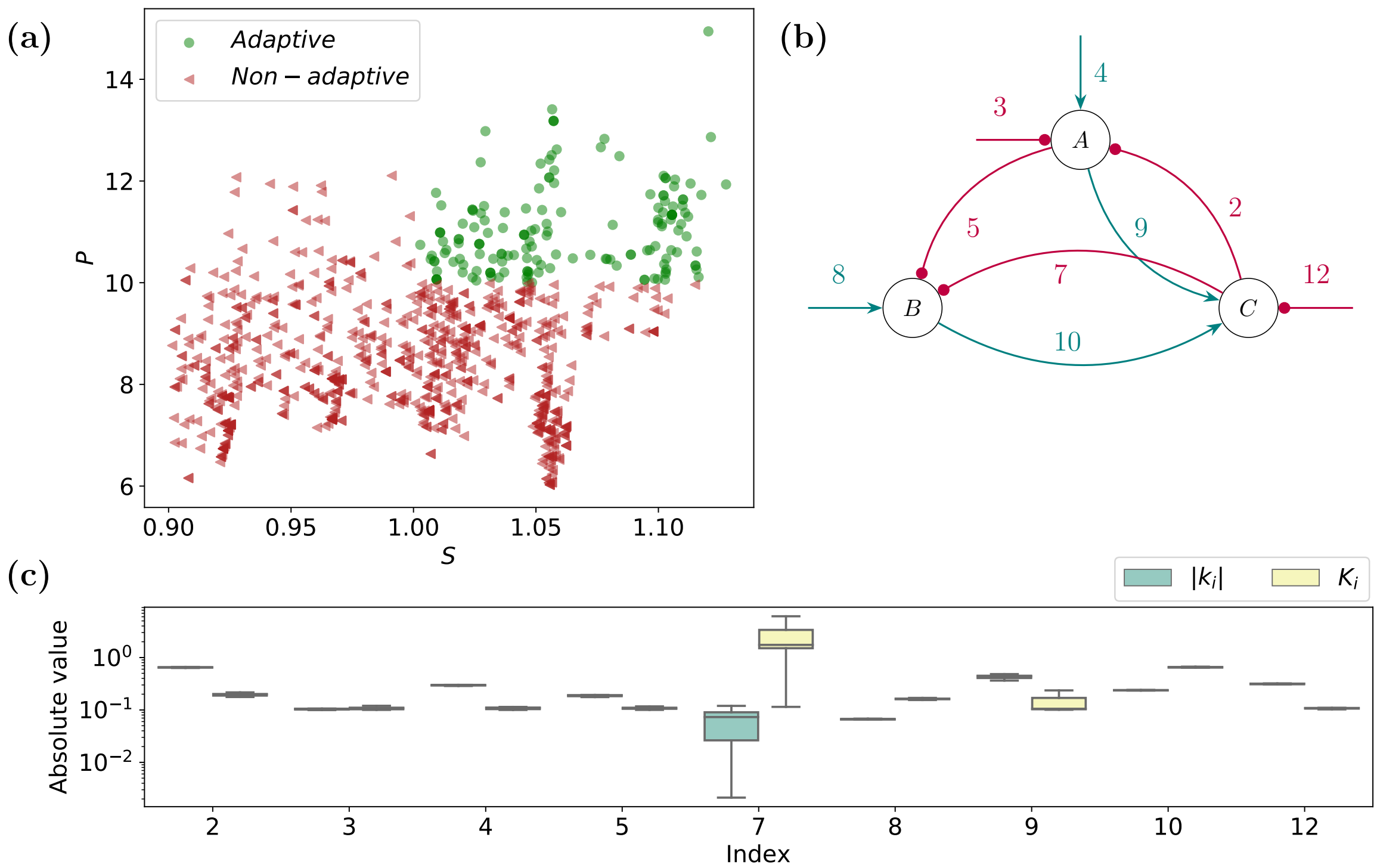

Figure S5: Pendant for Fig S4 for panel (p) in Fig S3. Again, not all parameter combinations lead to adaptation. Indeed, only a very narrow range of samples leads to adaptation.
